# Handling and Storage Procedures Have Variable Effects on Fatty Acid Content in Fishes with Different Lipid Quantities

**DOI:** 10.1371/journal.pone.0160497

**Published:** 2016-08-01

**Authors:** Martina D. Rudy, Martin J. Kainz, Martin Graeve, Stefanie M. Colombo, Michael T. Arts

**Affiliations:** 1 National Water Research Institute, Environment and Climate Change Canada, 867 Lakeshore Road, Burlington, ON, Canada; 2 WasserCluster–Biologische Station Lunz, Dr. Carl Kupelwieser Promenade 5, Lunz am See, Austria; 3 Alfred Wegener Institute, Helmholtz Centre for Polar and Marine Research, Am Handelshafen 12, Bremerhaven, Germany; 4 Department of Chemistry and Biology, Ryerson University, 350 Victoria Street, Toronto, ON, Canada; Universidade de Vigo, SPAIN

## Abstract

It is commonly assumed that the most accurate data on fatty acid (FA) contents are obtained when samples are analyzed immediately after collection. For logistical reasons, however, this is not always feasible and samples are often kept on ice or frozen at various temperatures and for diverse time periods. We quantified temporal changes of selected FA (μg FAME per mg tissue dry weight) from 6 fish species subjected to 2 handling and 3 storage methods and compared them to FA contents from muscle tissue samples that were processed immediately. The following species were investigated: Common Carp (*Cyprinus carpio*), Freshwater Drum (*Aplodinotus grunniens*), Channel Catfish (*Ictalurus punctatus*), Antarctic Eelpout (*Pachycara brachycephalum*), Rainbow Trout (*Oncorhynchus mykiss*) and Arctic Charr (*Salvelinus alpinus*). The impact of storage method and duration of storage on FA contents were species-specific, but not FA-specific. There was no advantage in using nitrogen gas for tissue samples held on ice for 1 week; however, holding tissue samples on ice for 1 week resulted in a loss of FA in Charr. In addition, most FA in Trout and Charr decreased in quantity after being stored between 3 and 6 hours on ice. Freezer storage temperature (-80 or -20°C) also had a significant effect on FA contents in some species. Generally, we recommend that species with high total lipid content (e.g. Charr and Trout) should be treated with extra caution to avoid changes in FA contents, with time on ice and time spent in a freezer emerging as significant factors that changed FA contents.

## Introduction

Analyzing the fatty acid (FA) content of aquatic organisms has become an important biochemical tool and is now widely used to evaluate food web interactions and the nutritional status of individuals and populations [1, 2, 3, 4]. In particular, aquatic ecologists now frequently employ FA as trophic biomarkers [5, 6] to quantify the flow of energy and nutrients in food webs and as key metrics to better understand and predict the growth, reproduction, and survivorship of individuals and populations [7, 8, 9, 10]. In addition, n-3 long-chain (i.e. ≥ 20 carbons) polyunsaturated FA (LC-PUFA) have garnered considerable scientific and public attention because of their benefits to animal and human health [11, 12, 13].

Research into the multiple uses of FA in the aquatic sciences is a rapidly growing field, with an exponentially increasing number of published papers; as of the end of 2014, >29,000 studies have been published that deal with at least some aspect pertaining to lipids and FA in marine and freshwater ecosystems (S1 Fig). Therefore, it is important to evaluate the efficacy of commonly used procedures for handling and storing organisms and/or tissues destined for FA analysis precisely because such procedures have the potential to cause biochemical modifications of FA. Such artifacts could well lead to false or misleading conclusions [14, 15, 16, 17]. For example, it is known that the enzymatic and chemical reactions that alter lipids continue post-mortem and may be different than those occurring prior to death [18]. Thus, in the ideal situation, lipids are extracted and subsequently derivatized to methyl esters immediately following collection. However, since it is usually unrealistic to perform lipid extractions in the field, the common practice is to store organisms on ice, keep them frozen at temperatures ranging from -20 to -80°C or in liquid nitrogen (-196°C), or immerse them in solvent(s) for periods ranging from hours to several years prior to lipid analysis (S2 Fig). Knowledge about the potential effects of different storage procedures on FA modification is therefore needed to ensure the accuracy and reliability of FA analyses [15]. Finally, from a human health perspective, the oxidation and hydrolysis of PUFA that may occur during improper storage procedures is undesirable and potentially limits the shelf-life of fish [19].

Lipid oxidation and hydrolysis rates during storage are species-, tissue-, and temperature-dependent [20, 21]. Oxidation is a normal process controlled by antioxidants in living tissues; however, this protective function can decrease significantly after death resulting in lipid degradation [18]. Thus, minimizing the sample’s exposure to oxygen while in storage (by, for example, substituting air with a dry nitrogen gas atmosphere) may be important. Lipids are also hydrolyzed by lipases that are more mobile in aqueous environments. Therefore, researchers often limit “available” water by freezing or lyophilizing (freeze-drying) tissue samples [20].

Among past studies, methods of aquatic organism sample handling and storage prior to extraction of lipids and their FA varied considerably or were not reported at all (S2 Fig). Of the 60 studies that we surveyed that reported FA analysis, 17% did not provide information on storage temperature or duration. In contrast, 13% of the studies mentioned that samples were kept on ice for several hours while in the field and then transferred to a -20°C freezer for long-term storage or were temporarily stored at -20°C and transferred to -80°C until they could be processed. The practice of flash-freezing samples in liquid nitrogen and transferring them to a -80°C freezer for long-term storage [22] was reported in 28% of the surveyed papers. Several authors also stored samples under nitrogen gas to prevent oxidative damage (S2 Fig). The alternate method of storing samples in solvents (e.g. CHCl_3_, 0.01% butylated hydroxytoluene) prior to lipid analyses is used by many researchers [17, 23], but was not evaluated in this study. Because different studies use diverse methods there is a need to evaluate and compare techniques to determine which are the most protective against lipid degradation.

Previous studies that addressed the possible effect of sample handling and storage prior to lipid extraction showed changes in lipid classes when samples were maintained at only moderate freezing temperatures (-1 to -20°C) for periods ranging from 3 months to several years [14–16, 22, 24]. For example, triacylglycerols and phospholipids were shown to degrade resulting in an increase of free fatty acids, monoacylglycerols and diacylglycerols in algae, zooplankton [15, 22] and fish [14, 21]. These findings point to lipase activity which causes degradation when samples are not immediately frozen in liquid nitrogen upon collection and stored at -80°C. In each of these studies researchers tested for temporal degradation of FA while in storage for a limited number of species (usually one). Therefore, there is a need to investigate how the same handling and storage protocols affect the FA content of aquatic species (in this case fishes) from different taxonomic families, from different environments (i.e., marine and freshwater) and that have, at the outset, different FA and total lipid contents in their tissues. Furthermore, there are numerous analytical methods routinely used for measuring lipid oxidation in tissues [25]; however, most of these studies focus on quantifying the products of oxidation rather than quantifying changes in FA contents, which is useful in investigating food web dynamics, biomarker interpretation, and evaluating nutritional quality.

Proper handling and storage of fish is also of commercial importance. For example, the duration of time spent on ice may affect the lipid and fatty acid quality prior to human consumption, thereby affecting the nutritional quantity of fish. Therefore, it is important, from a nutritional perspective, to quantify potential changes in total lipid and fatty acid contents in fish muscle tissue after they are caught, handled and stored prior consumption.

The objectives of the present study were to: a) determine the effect of commonly used handling methods (flash frozen, held on ice, presence of nitrogen gas) on FA content, b) determine the effect of storage temperature (-80 vs. -20°C) and time (up to 6 months) on FA content, and, c) investigate these methods on a variety of commercially important and taxonomically-diverse fish species, originating from different environments (from both freshwater and marine environments) and with different total lipid levels and FA contents in muscle tissue.

## Materials and Procedures

### Test species

We chose a variety of taxonomically diverse fish species available to the researchers involved in the study, from both freshwater and marine ecosystems, and with different total lipid levels and FA contents stored in the muscle tissue. The Common Carp *(Cyprinus carpio*, Linnaeus, 1758; hereafter Carp) is a freshwater omnivorus fish native to Asia that has been introduced to many parts of the world and is the most consumed fish worldwide [26]. The Freshwater Drum (*Aplodinotus grunniens*, Rafinesque, 1819; hereafter Drum) is a predator endemic to North and Central America that feeds mostly on invertebrates and mussels. The Channel Catfish (*Ictalurus punctatus*, Rafinesque, 1818; hereafter Catfish), an omnivore, is native to flowing waters in temperate environments in North America. The Antarctic Eelpout (*Pachycara brachycephalum*, Pappenheim, 1912; hereafter Eelpout) is an Antarctic Ocean predator. Rainbow Trout (*Oncorhynchus mykiss*, Walbaum, 1792; hereafter Trout) and Arctic Charr (*Salvelinus alpinus*, Linnaeus, 1758; hereafter Charr) are freshwater predatory salmonids native to tributaries of the Pacific Ocean in Asia and North America and to Arctic and some alpine lakes and tributaries to the Arctic Ocean, respectively. Carp, Trout, and Charr are also increasingly important for freshwater aquaculture.

### Sampling

Fish for our study were all caught in the summer of 2009 (at seasonal water temperatures for the respective temperate locations); Carp, Drum and Catfish were from Hamilton Harbour, Ontario, Canada (~20.2°C); Eelpout were from Antarctica but reared (at ~2°C) in Bremerhaven, Germany; Trout were from pre-alpine Lake Lunz (at ~10.3°C), and Charr were reared in an aquaculture facility located near Lake Lunz, Austria (11–14°C). Caught by local anglers, fish from Hamilton Harbour were humanely-euthanized by the anglers when landed, and immediately transferred to us. Since Trout and Charr from aquaculture were not exposed to any dietary threat, harm or genetic modification permits were not required from the Ethics Commission in Austria. After collection by professional fishermen, fish were rendered unconscious (blow on the head) and then killed by cardiac incision following the Federal Act on the Protection of Animals, Austria (http://www.ris.bka.gv.at) specifically for this research. Eelpout was euthanized by blow on the head, according to standard procedures in place at the Alfred Wegener Institute (Protocol 522-27-11/02-00 (93) valid 2008–2011). Eighty ~ 2 g skinless dorsal muscle plug (biopsy) samples (landmarked to either side of the dorsal fin) were quickly taken from a single fish from each species within minutes of collection. In order to directly test the effects of different storage protocols and storage times on FA, the 80 muscle plugs used for all treatments and replicates came from a single individual, for each species. Using multiple individuals would have introduced a new source of variation and bias in our investigation of analytical replicates [27]; therefore, to minimize effects of biological variation in order to allow a statistically-appropriate focus on the effect of storage, we used replicates from the same individual. Lipid composition within this specific region of dorsal muscle tissue (for example, see region P2 in [28]) is known to be homogenous, even in fish with high total lipid contents [28, 29, 30]; therefore, the muscle plugs can serve as appropriate replicates to compare among different sampling and storage protocols.

**Fig 1:**
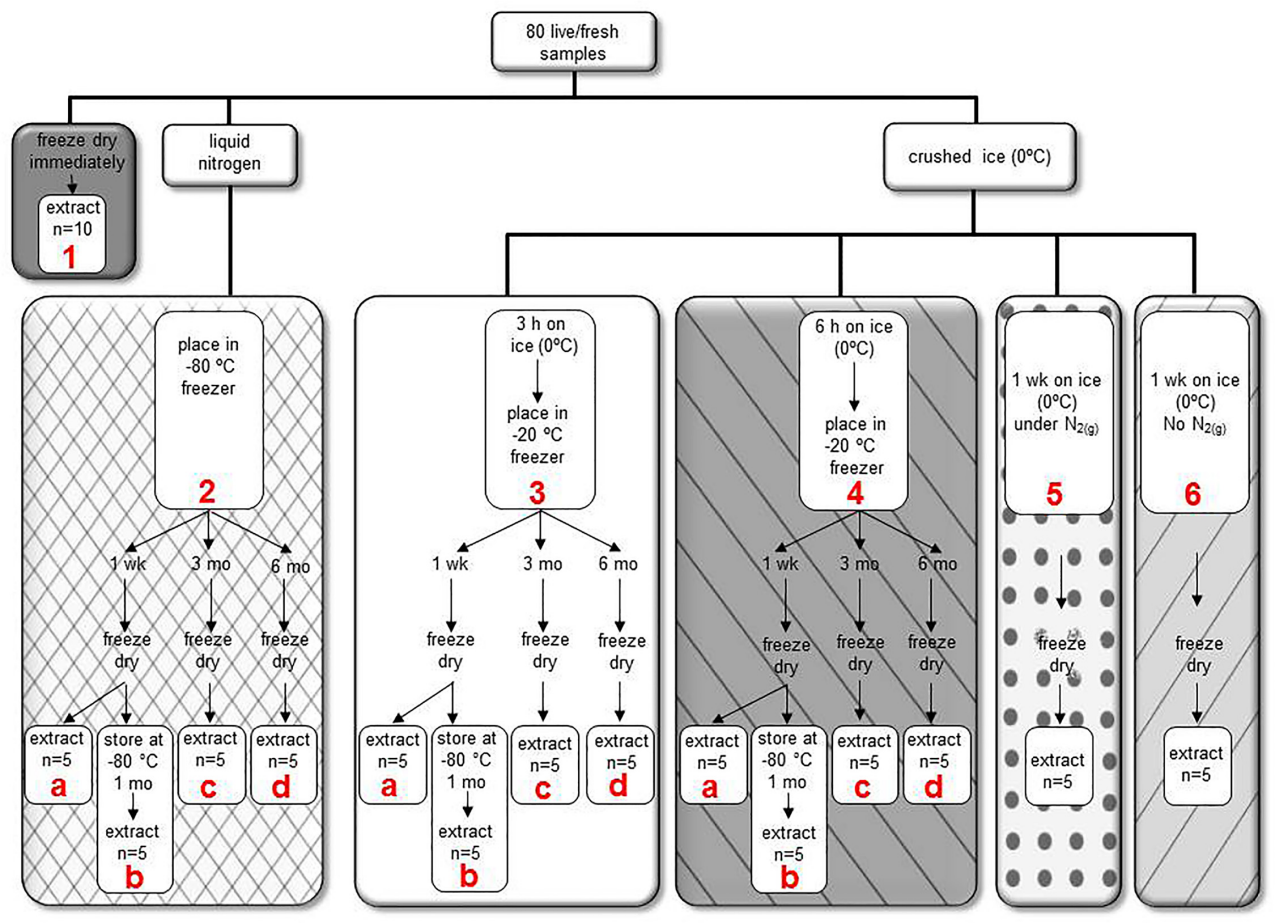
Experimental design and details of the handling and storage procedures used for each of the organisms under investigation. The initial control was freeze dried immediately (1), except for Arctic Charr and Rainbow Trout, where the control was flash frozen and stored at -80°C for one week (2a).

All tissue samples were freeze-dried immediately, frozen in liquid nitrogen, or placed in a cooler of crushed ice, then subsequently stored according to specific protocols (Fig 1). Our goal was to simulate some of the most commonly used handling and storage conditions observed in the literature, with the controls being either those samples that were processed immediately upon collection or frozen in liquid nitrogen and stored for 1 week at -80°C. The conditions tested were placement on ice for 3 or 6 h before storage at -20°C (Fig 1) following which these conditions were observed over time; samples were stored frozen for 1 week, 3 months or 6 months at their respective storage temperatures. Half the samples that were stored for 1 week were then freeze-dried and returned to storage for 1 month to investigate whether storing samples freeze-dried protected lipid and FA content. All samples were lipid extracted and analyzed for total lipid (gravimetric determination), and derivatized to FA methyl esters (FAME) and analyzed for FA content (gas chromatography with flame ionization detection). Fatty acids were reported as both mass fractions (i.e., weight per unit dry mass; S1 Table) and proportions of individual FA (S2 Table); however, the statistical analyses presented and discussed were on mass fraction data only (μg FAME per mg tissue dry weight), and are referred to as FA contents.

### Total lipid and fatty acid analysis

After the designated storage time, tissue samples were freeze-dried for as long as 4 d (depending on the tissue matrix) at –62°C under vacuum. The dried fish muscle was homogenized with a mortar and pestle, in liquid nitrogen, to a fine powder. All samples were weighed to the nearest microgram. The mass used in the extraction depended on the species examined. Total lipids were extracted using a modified Folch et al. method [31], as in McMeans et al. [32]. In brief, lyophilized tissue samples were extracted using 2 mL volumes of chloroform/methanol (2:1; v/v) three times and then pooled. The more polar impurities were removed by adding NaCl (0.9% in water); this layer was discarded following centrifugation. The resulting lipid containing solvent was concentrated to 2 mL and 2 aliquots (100 μL each) were removed and evaporated to dryness to determine the content of total lipids (i.e. mg per unit biomass, using gravimetric analysis). The gravimetric method is used routinely for determination of total lipid, and is also commonly used as a quality control check to verify completeness of extraction [33, 34] The lipid extract was then prepared for gas chromatography analysis by derivatizing FA to FAME using sulfuric acid as the catalyst [32]. Fatty acid methyl esters were extracted twice using hexane:diethyl ether (1:1; v/v) with butylated hydroxytoluene (BHT; 0.01%) used as a preservative and subsequently dried under a gentle stream of nitrogen. The dry FAME extract was re-dissolved in hexane and individual FAME were separated using a Hewlett Packard 6890 gas chromatograph (GC) and THERMO Trace GC (in Austria); in Germany as per [35] using an Agilent DB-FFAP column (DB-FFAP 30 m x 0.25 mmID, 0.25 μm film) and in Canada and Austria as in McMeans et al. [6] using a Sigma-Aldrich SP 2560 column. Both are highly polar capillary columns specifically designed for FA analysis. All solvents used in the extraction and FAME derivatization procedures were of high purity HPLC grade (>99%). FAME in samples were identified by comparison of their retention times with a known standard (Supelco 37-component FAME standard, 47885-U; and docosapentaenoic acid methyl ester, Supelco, 47563-U) and quantified with a 5-point calibration curve using the same standards. A known concentration of 5 alpha cholestane (Sigma C8003) was added to each sample prior to extraction to act as a surrogate internal standard to measure method and instrument recovery. In addition, 5 alpha cholestane is a saturated 27-carbon steroid precursor and its chromatographic response does not interfere with any FA in our chromatograms, therefore, we assume proportionate standard/FA losses. Cholestane is commonly used as an internal standard for fatty acid and sterol analysis in plant and animal lipids [36, 37], including fish [38]. For tissue samples analyzed in Germany (Eelpout), 23:0 FAME was used as the internal standard. Extraction efficiency was calculated by comparing the amount of internal standard (cholestane or 23:0) added to the sample relative to the amount recovered in the FAME, as determined by GC. For all investigated organisms, FA contents reported here included only those that had a mass fraction >1 μg FA · mg^-1^ dry weight, with the exception of Eelpout where mass fractions are reported on a wet weight basis.

### Statistical analyses

Total lipid and FA contents (μg FAME mg^-1^ dry weight tissue, square root transformed) were compared amongst storage methods for each species separately. A t-test was used to determine the difference in FA content between storing with or without nitrogen gas, while stored on ice for 1 week. After confirmation that there was no significant difference between storing under nitrogen gas conditions, these two treatments were pooled for each species to form a single group which were stored on ice for 1 week (regardless of nitrogen gas). A t-test was then used to compare this group with the initial control (or the flash frozen, stored for 1 week when the initial control was not available). A nested ANOVA was used to test the main effect of handling method (either on stored ice for 3 or 6 hours prior to freezing) and the nested effect of time (1 week, 1, 3, and 6 months) within handling method. In species where there was no significant difference between storing on ice for 3 versus 6 hours, these treatments were pooled (i.e. 3 and 6 hours storage was pooled) and compared to the flash frozen method with freezer storage at -80°C. This nested ANOVA was designed to determine whether there is a difference between storing tissue samples in a freezer at -80 or -20°C, and whether the duration of time within either of these methods significantly changed FA content. For each model tested, the residuals were examined to evaluate the appropriateness of the model, therefore normality, homogeneity, and independence of residuals were considered. Differences were considered significant at p<0.05 for all tests. If a p-value was close to 0.05 and residuals were not normal, a p-randomization was conducted >5000 times to test the data empirically. All analyses were conducted using Minitab Statistical Software (Version 16, State College, PA).

In addition, multivariate statistical analyses (conducted in PRIMER-E Ltd., Plymouth, UK, ver. 7) were used to help illustrate the cumulative effect of storage method and time on tissue fatty acid content, including principal components analysis (PCA), principal coordination analysis (PCO), multi-dimensional scaling (MDS), analysis of similarities (ANOSIM), similarities in percentages (SIMPER), and distance-based linear modelling (DistLM).

## Results

### Total lipid and fatty acids

The mass fraction FA data and proportion (percent) FA and total lipid data are reported in S1 and S2 Tables. Statistical analyses of the mass fraction FA data can be found in Tables 1–3. Assessment and discussion are in reference to FA content (μg FAME per mg tissue dry weight) only.

**Table 1:** The effect of storing tissue samples on ice for one week on fatty acid contents (μg mg^-1^ dry weight) compared with the control, presented as p-values from pairwise t-tests.

Fatty acid	Carp	Drum	Catfish	Eelpout	Trout	Charr
16:0	0.514	0.303	0.336	0.427	**0.047**	**0.004**
16:1n-7	0.419	0.280	0.336	0.288	0.073	**0.005**
18:0	0.318	0.812	0.864	0.521	0.075	**0.005**
18:1n-9	0.501	0.137	0.344	0.571	0.101	**0.006**
18:1n-7	0.570	0.944	0.238	0.617	0.093	**0.006**
18:2n-6	0.507	0.277	0.288	0.319	0.109	**0.005**
18:3n-3	0.519	0.605	0.576	0.387	0.107	**0.005**
20:4n-6	0.408	0.395	0.379	0.925	0.054	**0.009**
20:5n-3	0.598	0.125	0.225	0.728	0.073	**0.007**
22:5n-3	0.938	0.754	0.221	0.619	0.182	**0.007**
22:6n-3	0.164	0.738	0.521	0.721	0.142	**0.018**
∑n-3	0.535	0.481	0.272	0.068	0.110	**0.011**
∑n-6	0.385	0.954	0.263	0.087	0.106	**0.006**
Total lipid	0.703	0.895	0.429	0.683	0.080	**0.008**

**Table 2:** Effect of temporary storage time on ice (3 or 6 hours; df = 1, 32) and duration of time spent in freezer storage at -20°C (1, 3, or 6 months; df = 6, 32) on fatty acid contents (μg mg^-1^ dry weight), presented as p-values from nested ANOVA.

Fatty acid	Storage method	Time in freezer (nested factor)
**Carp**		
16:0	0.096	0.129
16:1n-7	0.100	0.111
18:0	0.062	0.089
18:1n-9	0.087	0.138
18:1n-7	0.077	0.092
18:2n-6	0.097	0.129
18:3n-3	0.087	0.113
20:4n-6	0.371	0.061
20:5n-3	0.156	0.219
22:5n-3	0.092	0.174
22:6n-3	0.081	0.007
∑n-3	0.090	0.106
∑n-6	0.107	0.177
Total lipid	0.068	0.187
**Drum**		
16:0	**0.014**	**< 0.001**
16:1n-7	0.146	**< 0.001**
18:0	0.045	**< 0.001**
18:1n-9	0.059	**< 0.001**
18:1n-7	0.078	**< 0.001**
18:2n-6	0.446	**< 0.001**
18:3n-3	0.713	**< 0.001**
20:4n-6	0.087	**< 0.001**
20:5n-3	0.147	**< 0.001**
22:5n-3	0.563	**< 0.001**
22:6n-3	0.524	**< 0.001**
∑n-3	0.163	**0.004**
∑n-6	0.210	**0.004**
Total lipid	0.250	**< 0.001**
**Catfish**		
16:0	0.196	**0.001**
16:1n-7	0.286	0.611
18:0	0.223	0.694
18:1n-9	0.256	0.296
18:1n-7	0.347	0.607
18:2n-6	0.284	0.629
18:3n-3	0.304	0.653
20:4n-6	0.264	0.827
20:5n-3	0.273	0.820
22:5n-3	0.300	0.655
22:6n-3	0.395	0.877
∑n-3	0.297	0.769
∑n-6	0.093	0.793
Total lipid	**0.046**	0.609
**Eelpout**		
16:0	0.124	0.391
16:1n-7	0.103	0.400
18:0	0.087	0.169
18:1n-9	0.152	0.595
18:1n-7	0.052	0.408
18:2n-6	**0.015**	0.272
18:3n-3	0.144	0.342
20:4n-6	0.200	0.110
20:5n-3	0.049	0.152
22:5n-3	**< 0.001**	**0.044**
22:6n-3	**0.009**	**0.030**
∑n-3	**0.015**	0.119
∑n-6	**0.039**	0.298
Total lipid	**0.030**	0.432
**Trout**		
16:0	**< 0.001**	0.794
16:1n-7	**< 0.001**	0.788
18:0	**< 0.001**	0.830
18:1n-9	**< 0.001**	0.834
18:1n-7	**< 0.001**	0.825
18:2n-6	**< 0.001**	0.857
18:3n-3	**< 0.001**	0.857
20:4n-6	**< 0.001**	0.869
20:5n-3	**< 0.001**	0.857
22:5n-3	**< 0.001**	0.811
22:6n-3	**< 0.001**	0.931
∑n-3	**< 0.001**	0.894
∑n-6	**< 0.001**	0.855
Total lipid	**< 0.001**	0.921
**Charr**		
16:0	**0.036**	**0.007**
16:1n-7	0.052	**0.004**
18:0	**0.034**	**0.008**
18:1n-9	**0.048**	**0.004**
18:1n-7	**0.040**	**0.005**
18:2n-6	**0.039**	**0.006**
18:3n-3	**0.040**	**0.006**
20:4n-6	0.052	**0.003**
20:5n-3	**0.045**	**0.003**
22:5n-3	0.073	**0.005**
22:6n-3	0.109	**0.003**
∑n-3	0.070	**0.004**
∑n-6	**0.040**	**0.005**
Total lipid	**0.023**	**0.014**

**Table 3:** Effect of freezer storage temperature on fatty acid contents (μg mg^-1^ dry weight), either at -80°C or -20°C (pooled groups from ice storage at 3 and 6 hours; df = 2, 52), and duration of time spent in freezer (nested factor; df = 6, 52), presented as p-values from nested ANOVA.

	Carp		Catfish		Drum		Eelpout	
Fatty Acid	Freeze Temperature	Time	Freeze Temperature	Time	Freeze Temperature	Time	Freeze Temperature	Time
16:0	0.185	0.174	0.411	0.426	**0.008**	**< 0.001**	0.101	0.100
16:1n-7	0.162	0.128	**0.031**	0.339	0.185	**< 0.001**	0.130	0.186
18:0	0.110	0.360	**0.046**	0.480	**0.006**	**< 0.001**	0.939	**0.007**
18:1n-9	0.189	0.217	**0.037**	0.314	**0.025**	**< 0.001**	**0.048**	0.478
18:1n-7	0.164	0.179	0.107	0.332	**0.006**	**< 0.001**	0.198	0.242
18:2n-6	0.200	0.216	0.061	0.363	**0.004**	**< 0.001**	0.676	0.111
18:3n-3	0.199	0.175	0.056	0.378	**0.001**	**< 0.001**	0.949	0.106
20:4n-6	0.113	**0.006**	0.222	0.641	**0.001**	**< 0.001**	**0.034**	**0.005**
20:5n-3	0.137	0.258	0.212	0.697	**0.001**	**< 0.001**	**0.007**	**0.043**
22:5n-3	0.362	0.524	0.080	0.397	**0.001**	**< 0.001**	0.413	0.339
22:6n-3	0.086	**0.002**	0.170	0.724	**< 0.001**	**< 0.001**	**0.002**	**0.004**
∑n-3	0.173	0.174	0.141	0.563	**0.017**	**0.020**	**0.025**	**0.038**
∑n-6	0.148	0.127	0.378	0.389	0.125	0.062	0.393	0.086
Total lipid	0.221	0.391	0.236	0.379	**< 0.001**	**< 0.001**	0.083	**0.015**

### Effect of nitrogen gas on samples held on ice for 1 week

**Fig 2:**
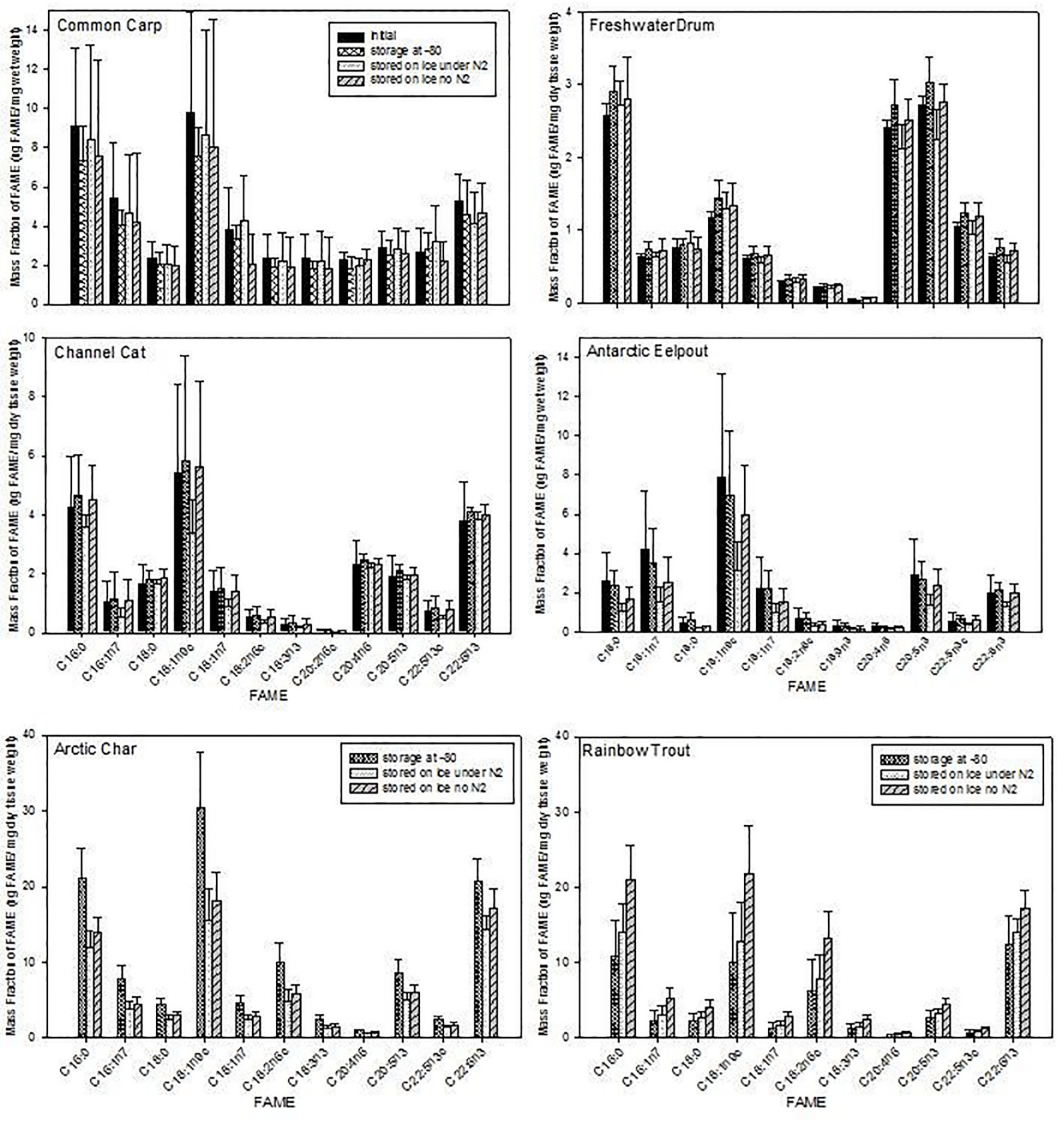
Mean and standard deviations of FAME (μg FAME/mg dry tissue weight). Six species of fish for four handling treatments (initial, 1 week at -80°C, 1 week on ice with or without nitrogen gas). For Arctic Char and Rainbow Trout, the control is flash frozen and stored for 1 week at -80°C.

There was no significant difference in the FA contents of tissue samples (except trout) held on ice for 1 week with nitrogen gas compared to those without nitrogen gas (Fig 2). Trout was the only exception, as all FA contents were significantly higher for the treatment without nitrogen gas present.

### Effect of keeping samples on ice for 1 week compared to control

Keeping tissue samples on ice for 1 week (pooled groups with and without nitrogen atmosphere) generally did not affect the FA contents in comparison to the initial control for most fish species, with the exception of Charr (Table 1; Fig 2). In Charr, all FA and total lipid contents were significantly higher in the control than the ice storage treatment.

### Effect of holding time on ice (3 or 6 hours) and storage time at -20°C

**Fig 3:**
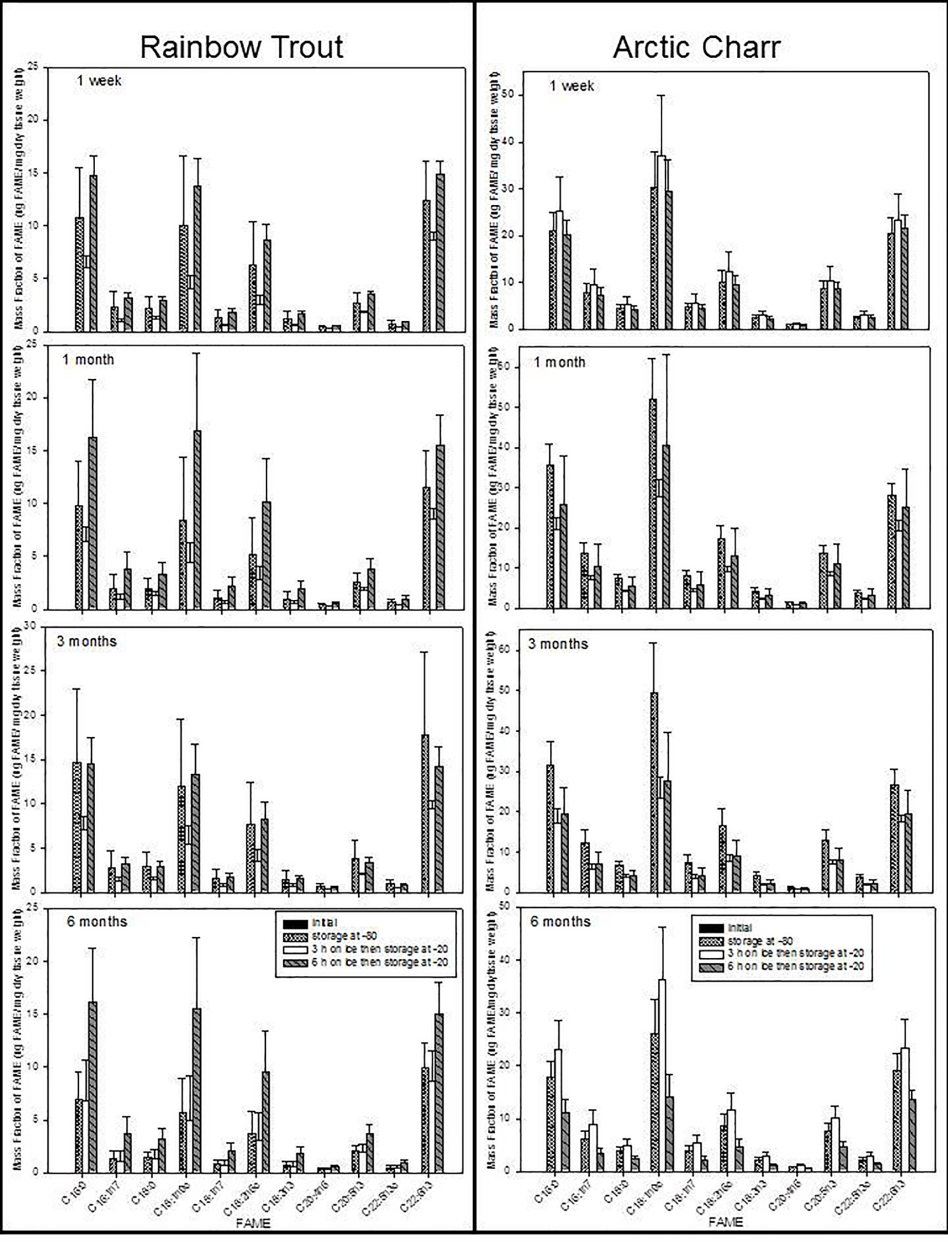
Mean and standard deviations of FAME (μg FAME/mg dry tissue weight) of Rainbow Trout and Arctic Charr for the four storage treatments. The initial control is 1 week storage at -80°C, and 3 or 6 hours on ice treatments at increasing storage time (1 week, 3 or 6 months) in a -20°C freezer.

**Fig 4:**
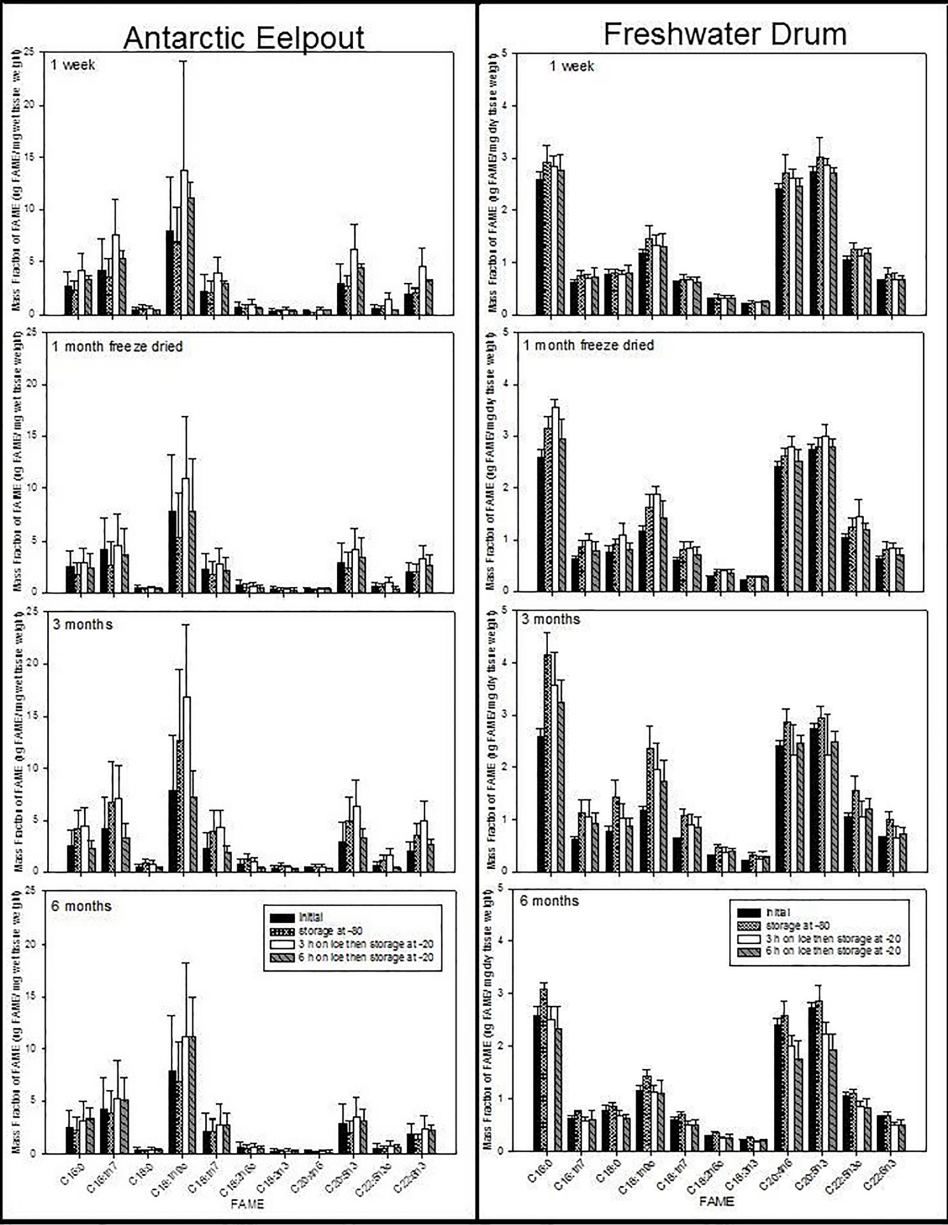
Mean and standard deviations of FAME (μg FAME/mg dry tissue weight) of Eelpout and Freshwater Drum. Four storage treatments (initial, 1 month, 3 or 6 hours on ice) at increasing storage time (1 week, 3 or 6 months) in a -20°C freezer.

**Fig 5:**
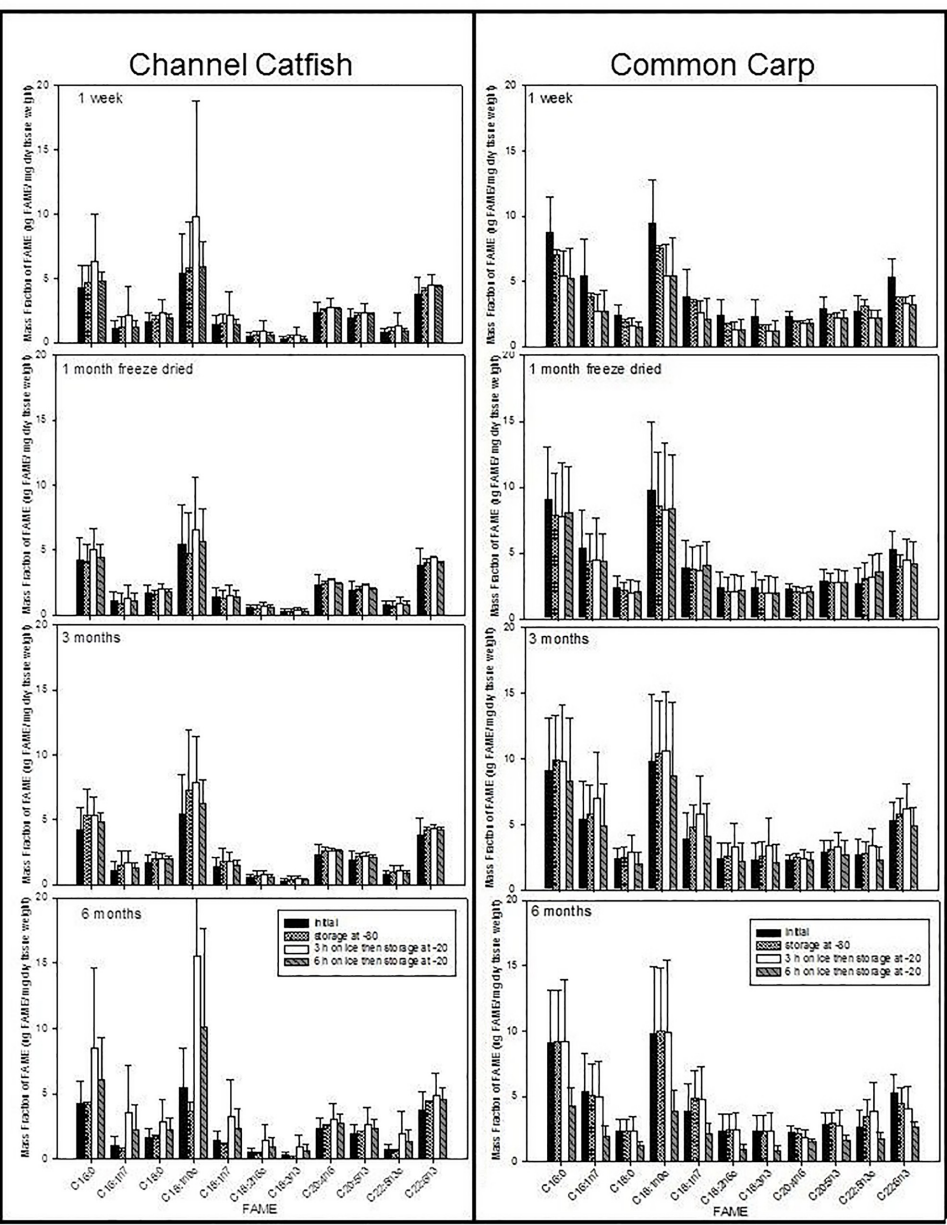
Mean and standard deviations of FAME (μg FAME/mg dry tissue weight) of Channel Catfish and Carp. Four storage treatments (initial, 1 month, 3 or 6 hours on ice) at increasing storage time (1 week, 3 or 6 months) in a -20°C freezer.

Holding time on ice and storage time at -20°C affected FA contents depending on species (Table 2; Figs 3–5). For Trout and Charr, total lipid and FA contents were significantly different depending on holding time on ice (3 or 6 hours) (Table 2; Fig 3). For Carp and Catfish, FA contents did not differ depending on holding time on ice, except for total lipid in Catfish (Table 2; Fig 4). For Eelpout and Drum, this was dependent on the FA, because in Eelpout, the contents of 18:2n-6, 22:5n-3, 22:6n-3, the sums of n-3 and n-6 fatty acids, and total lipid, and 16:0 in Drum, were significantly different depending on holding time on ice, but all other FA did not differ among treatments (Table 2; Fig 5). For Charr and Eelpout, FA contents were higher when held on ice for 3 hours compared to 6 hours, but varied depending on treatment (Table 2). For Charr (Fig 3) and Drum (Fig 4), the duration of time spent in a -20°C freezer was a significant nested factor within holding time spent on ice prior to freezer storage (Table 2). FA contents tended to decrease over time from 1 week to 3 months, but did not decrease after 6 months of freezer storage.

### Effect of freezer storage temperature (-20°C or -80°C) and time of storage

In fish where the FA contents were not different depending on the number of hours held on ice prior to freezer storage, the 3 and 6 hours treatments were pooled within their respective freezer storage times (up to 6 months). The pooling of the 3 and 6 hours storage on ice treatments allowed for the comparison between freezer storage at -20°C (3 and 6 hour pooled) and -80°C. The FA contents in Drum differed significantly between -20 and -80°C, and time of freezer storage was also significant factor that affected FA contents (Table 3). FA contents were higher when stored at -80°C than at -20°C for Drum. Eelpout also showed a significant difference between storing at -20 and -80°C; however, this was only the case for a few FA. Storing at -20 or -80°C for any period of time generally did not affect FA contents in Carp or Catfish.

Multivariate statistical analyses did not help distinguish differences in FA content depending on storage method and duration; however, not surprisingly, the FA content of the different fishes were significantly different according to PERMANOVA (p < 0.01).

## Discussion

We conclude that the best possible scenario of the methods we tested to prevent changes in total lipids and FA content (μg FAME per mg tissue dry weight) is immediate freezing at -80°C. This conclusion agrees with other researchers who also suggested that tissue samples be frozen at -80°C upon collection or as soon as possible after sampling or death [22, 27]. However, we show that, for most fish species, deviations from this “gold standard” handling and storage conditions may also be possible without serious consequences to the FA content of the total lipid pool. Generally, the impact of storage method and duration of storage on FA content was species-specific, and depended on the total lipid content of the organism. Regardless of storage treatment, the FA content of fish was different depending on species, which appears to influence susceptibility to FA degradation. The effect of storage condition was greatest in Charr, which had the highest total lipid content (19% under control conditions) and Trout (11% under control conditions), while species with lower total lipid (<10% total lipid) were less affected, i.e., the storage conditions were not as critical. The lipid content not only relates to taxonomic classification, but also to the environment (i.e., marine or freshwater), geographic location and/or season (i.e., warm or cool waters), and distribution lipid storage; all of which may influence tissue FA content [39], and thus susceptibility to FA degradation upon various storage conditions.

The fish were subjected to several different handling method manipulations after sampling (flash frozen in liquid nitrogen, or stored on ice for 3 or 6 hours, or 1 week) and stored in freezers at different temperatures (-20 or -80°C) for increasing time periods. All tissue samples were freeze-dried prior to lipid extraction. Freeze-drying has been a recommended pre-treatment before long-term sample storage because the removal of water from tissues largely immobilizes lipases thus protecting lipids from enzymatic activity, particularly during the extraction process [40, 41]. Freeze-drying may also protect against degradation by minimizing freeze-thaw induced cell lysis [42]. However, samples have been stored for different time periods at different temperatures before being freeze-dried (see Methods). Thus, detected differences of FA contents in samples may have been subjected to a series of metabolic/catabolic reactions that caused FA to decrease in quantity, but such specific processes were not examined here. Further, because we analyzed tissue samples (i.e. muscle plugs) with relatively high surface: volume ratios, the samples we analyzed may be more susceptible to oxidation compared with whole fish or intact fillets (as would be used commercially). Therefore, the recommendations for the best handling and storage scenarios made based on our results may be conservative compared to the storage conditions required for whole fish in particular.

### Holding on ice for 1 week, with or without nitrogen gas

In general, it appeared that Charr and Trout were the most susceptible to changes in their FA contents as a result of compromised handling conditions. The most extreme handling method in this study, tested as a ‘worst case scenario’ was when muscle tissue samples were held on ice for 1 week, with or without the presence of nitrogen gas in the atmosphere of the sample. The presence of nitrogen gas did not appear to protect the FA content of tissue samples when stored on ice for a week. Therefore, in extreme situations when freezer storage is not immediately available, flushing samples in a nitrogen atmosphere may not help to prevent FA degradation. However, keeping tissue samples on ice for one week significantly altered the FA content for Charr (Table 1), resulting in a loss in FA content. There was no obvious pattern of degradation for certain FA; for example, PUFA were not observed to be more susceptible to degradation than SFA. Rather, the entire FA content was affected. Most FA significantly decreased in quantity after 1 week on ice compared to the control, as a result of the breakdown of FA and the formation of low molecular weight carbonyl compounds [43]. Therefore, we conclude that it is not advisable to keep samples on crushed ice for 1 week prior to analysis because significant changes to the FA content of organisms are expected, especially for species with total lipid ~10% or greater.

### Holding on ice for 3 or 6 hours

Less extreme handling methods (i.e., storing tissues on ice for 3 or 6 hours) did not affect the FA contents for some species, but significantly altered the FA contents in others (Table 2). Most FA contents in Trout and Charr were different after 6 compared to 3 hours storage on ice; however, FA in Carp and Catfish did not change after 6 hours on ice. Generally, species that were most significantly affected had higher total lipid contents, and would likely be more susceptible to FA degradation. Therefore, keeping samples on ice for up to 6 hours is detrimental for some fish species, likely as a function of total lipid content (see below). FA contents were generally higher when samples were held on ice for 3 hours, and decreased after 6 hours, indicating a loss of FA between 3 and 6 hours. This was not a function of the FA type (saturated vs. unsaturated), but rather the entire FA content was affected. However, regardless of the number of hours that tissue samples were held on ice, the time spent in a -20°C freezer after handling significantly affected the FA content in Char and Drum. FA contents tended to decrease over time in freezer storage from 1 week to 3 months, but did not decrease further after 3 months of freezer storage. Therefore, if tissue samples are stored at -20°C, it is advisable to analyze the FA content soon after organisms or tissues are sampled, as FA contents can significantly change with time.

### Freezer temperature

Freezer storage temperature (-80 or -20°C) also had a significant effect on the FA contents in some species (Table 3). Those organisms that did not show a difference in FA contents after being held on ice for either 3 or 6 hours were pooled to compare freezer storage at -20 and -80°C. Of these species, Drum showed a significant difference between these storage temperatures, with significant deterioration at -20°C compared to -80°C. In addition, the FA content significantly deteriorated over time for both freezer methods. Eelpout and Catfish showed changes in a few FA contents depending on storage temperature; however, they were not necessarily affected by the duration of time in the freezer, and this also did not seem to depend on FA type (saturated vs. unsaturated). Accelerated FA degradation at -20°C freezer storage has also been observed in other tissues, such as mammalian blood [27, 44]. Therefore, we conclude that the best practice is to store samples at -80°C if possible (in preference to -20°C).

### FA susceptibility to degradation

Long-chain PUFA are commonly thought to be more susceptible to degradation due to the methylene bridges between double bonds, which have particularly reactive hydrogens that are easily abstracted by free radicals [25]. It has thus been assumed that there should be preferential degradation of LC-PUFA relative to other lipid classes in improperly stored samples. However, we did not observe a specific FA, or group of related FA, to consistently and preferentially change quantity with increasingly poor handling and storage conditions across all species, though often a loss in several FA contents over time was observed. FA degradation was more of a function of species rather than FA type, which suggests species-specific FA dynamics during storage, likely as a function of total lipid in that species. Such observed alterations of FA in different organisms may depend on several factors such as lipid content, size, sex, diet, season, state at the moment of capture, importance and nature of the microbial load, veracity, state, number of lipases and how they are sequestered (or not) in cells and tissue type [21, 45].

### Implications

The effect of sample handling and storage on the FA contents of aquatic organisms is of particular interest to fisheries (for direct human consumption or commodities such as fish meal and oil), aquaculture, and ecology related fields, and sample handling and storage methods prior to lipid analyses can impact the interpretation and conclusion of results generated in these fields. It is well known that the content of total lipid in fish muscle varies among species, ranging from lean fish with < 2% of total lipid to fatty species that contain between 8 and 20% total lipid on a wet weight basis [21]. This is important because fatty fish may be particularly susceptible to lipid oxidation, as inferred in this study and others [21, 46]. The effect of storage temperature on lipid degradation is minimal and not systematic in lean fish species, such as Atlantic Cod (*Gadus morhua*) [14]. Similarly, Silver Carp (*Hypophthalmichthys molitrix*) that was held at room temperature for 8 d to mimic the extreme of fish market conditions did not demonstrate changes in FA content [47]. Fatty fish species in this study, especially Charr, were more susceptible to changes in FA contents as a consequence of handling and storage; however, certain FA or groups of FA (such as PUFA and n-3 FA) were not necessarily more sensitive to degradation. The susceptibility of certain species to degradation and changes in FA content may also be a function of lipid class composition, rather than total lipid content alone; however, this was beyond the scope of our study. However, FA are known to be affected by peroxidation regardless of whether they are present as free FA, or in triacylglycerols, diacylglycerols, monoacylglycerols or phospholipids [25].

The handling and storage procedures investigated here had variable effects on the FA content of total lipids in the fish species examined here. Certain species were more susceptible to lipid degradation, rather than certain FA (i.e. PUFA) that are known to be sensitive to oxidation. Generally, we found that species with high total lipid content (particularly Charr and Trout) should be treated with extra caution to avoid changes in the FA contents, with time on ice and time spent in a freezer emerging as significant factors that changed FA contents. Time spent in a freezer, either at -80°C or -20°C affected the FA contents of most species in this study, with the exception of Catfish and Carp which did not appear to be affected by the handling and storage manipulations in this study. We conclude that, in the best-case scenario, and especially for high lipid content species, it is important, when collecting aquatic organisms for lipid quantification or consumption, to store muscle tissue samples in a -80°C freezer and analyze them as soon as possible to avoid changes in FA contents.

## Supporting Information

S1 Fig Number of ISI-indexed journal publications, taken from Web of Science®, from 1990 to 2013. Search criteria was, 'lipid*' or 'fatty acid*' and either “lake or pond or stream or wetland or river” (in the case of freshwater ecosystems) or “ocean or sea or marine or estuary” (in the case of marine ecosystems). The dataset was subsequently refined to the, a) “Science and Technology” research domain, b) “article” document type and, c) following research areas = “environmental sciences ecology”, “fisheries”, “marine freshwater biology”, and “oceanography”.
(TIF)

S2 Fig Journal article survey of storage conditions used for various aquatic organisms. (TIF)

S1 Table Fatty acid content (μg FAME/mg dry tissue weight) of 6 fish species for all storage treatments. (DOCX)

S2 Table Mean and standard deviations of FAME proportions (%) of 6 fish species for all storage treatments. (DOCX)
