# Supplementary figures and images for: Handling and Storage Procedures Have Variable Effects on Fatty Acid Content in Fishes with Different Lipid Quantities
Source: PLoS One. 2016 Aug 1;11(8):e0160497. doi: 10.1371/journal.pone.0160497 (PMC4968796; doi:10.1371/journal.pone.0160497)

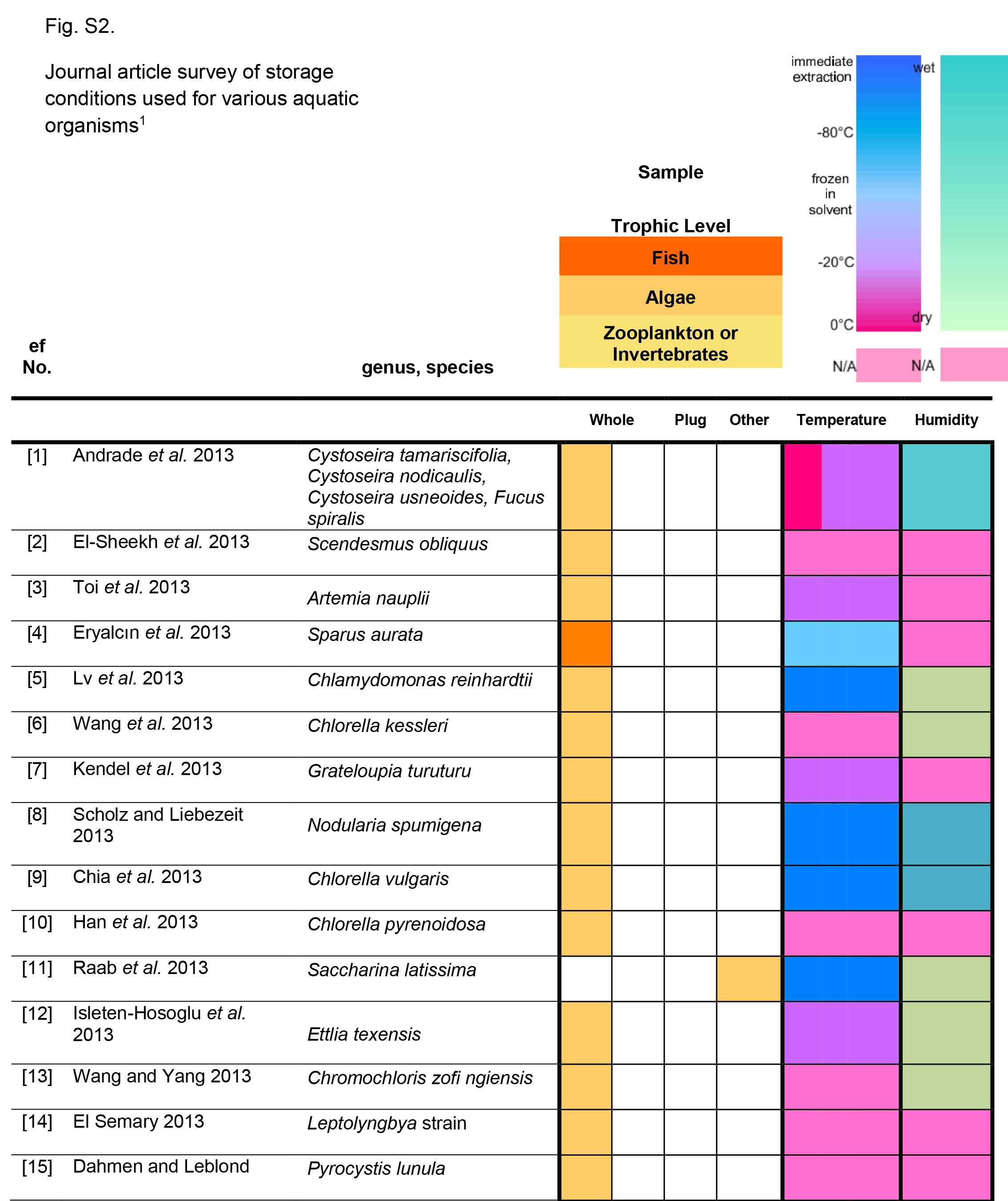

Supplement: S2 Fig — (TIF) [file pone.0160497.s002.tif]
