# Supplementary material for: Handling and Storage Procedures Have Variable Effects on Fatty Acid Content in Fishes with Different Lipid Quantities
Source: PLoS One. 2016 Aug 1;11(8):e0160497. doi: 10.1371/journal.pone.0160497 (PMC4968796; doi:10.1371/journal.pone.0160497)
Supplement: S1 Table — (DOCX) [file pone.0160497.s003.docx]

S1 Table. Fatty acid content (µg FAME/mg dry tissue weight) of 6 fish species for all storage treatments.

| ***Carp*** | | **Storage at -80°C** | | | | **Ice for 3 h then storage at -20°C** | | | | **Ice for 6 h then storage at -20°C** | | | | **On ice + N_2_** | **On ice – N_2_** |
| --- | --- | --- | --- | --- | --- | --- | --- | --- | --- | --- | --- | --- | --- | --- | --- |
| **Fatty Acid** | **Initial** | **1 week** | **1 month** | **3 months** | **6 months** | **1 week** | **1 month** | **3 months** | **6 months** | **1 week** | **1 month** | **3 months** | **6 months** | **1 week** | **1 week** |
| 16:0 | 9.1 ± 4.0 | 7.3 ± 1.8 | 7.8 ± 3.2 | 9.9 ± 3.4 | 9.2 ± 3.9 | 5.5 ± 1.9 | 7.8 ± 4.0 | 11.9 ± 5.9 | 9.2 ± 4.7 | 5.2 ± 2.3 | 8.0 ± 3.5 | 8.3 ± 2.9 | 4.3 ± 1.4 | 8.4 ± 4.8 | 7.6 ± 4.9 |
| 16:1n-7 | 5.4 ± 2.9 | 4.1 ± 0.8 | 4.4 ± 2.1 | 5.8 ± 2.2 | 5.0 ± 2.5 | 2.7 ± 1.2 | 4.5 ± 3.1 | 7.0 ± 3.5 | 5.0 ± 2.8 | 2.7 ± 1.6 | 4.3 ± 2.2 | 4.8 ± 3.3 | 1.9 ± 0.9 | 4.6 ± 3.0 | 4.2 ± 35 |
| 18:0 | 2.4 ± 0.9 | 2.1 ± 0.6 | 2.2 ± 0.6 | 2.5 ± 0.8 | 2.4 ± 0.9 | 1.6 ± 0.5 | 2.0 ± 0.9 | 2.9 ± 1.3 | 2.4 ± 1.1 | 1.4 ± 0.5 | 2.1 ± 0.8 | 2.0 ± 0.9 | 1.2 ± 0.3 | 2.0 ± 1.0 | 2.0 ± 1.0 |
| 18:1n-9 | 9.8 ± 5.2 | 7.6 ± 1.5 | 8.6 ± 4.1 | 10.4 ± 4.1 | 9.9 ± 4.9 | 5.5 ± 2.4 | 8.2 ± 5.1 | 13.2 ± 7.2 | 9.9 ± 5.5 | 5.4 ± 3.0 | 8.3 ± 4.2 | 8.7 ± 5.6 | 3.9 ± 1.6 | 8.6 ± 5.4 | 8.0 ± 6.5 |
| 18:1n-7 | 3.8 ± 2.1 | 3.4 ± 0.7 | 3.7 ± 1.7 | 4.8 ± 1.7 | 4.9 ± 2.1 | 2.6 ± 0.9 | 3.6 ± 2.0 | 5.8 ± 2.9 | 4.8 ± 2.5 | 2.1 ± 1.6 | 4.0 ± 1.9 | 4.1 ± 2.4 | 2.1 ± 0.8 | 4.2 ± 2.4 | 2.6 ± 1.3 |
| 18:2n-6 | 2.4 ± 1.2 | 1.9 ± 0.4 | 2.1 ± 1.1 | 2.6 ± 1.0 | 2.4 ± 1.2 | 1.3 ± 0.6 | 2.1 ± 1.3 | 3.3 ± 1.8 | 2.4 ± 1.4 | 1.3 ± 0.7 | 2.1 ± 1.1 | 2.2 ± 1.4 | 0.9 ± 0.4 | 2.2 ± 1.5 | 1.9 ± 1.6 |
| 18:3n-3 | 2.3 ± 1.2 | 1.8 ± 0.4 | 1.9 ± 1.0 | 2.5 ± 1.1 | 2.3 ± 1.2 | 1.2 ± 0.5 | 2.0 ± 1.3 | 3.4 ± 2.1 | 2.3 ± 1.4 | 1.2 ± 0.7 | 2.0 ± 1.1 | 2.1 ± 1.5 | 0.8 ± 0.4 | 2.2 ± 1.5 | 1.9 ± 1.6 |
| 20:4n-6 | 2.3 ± 0.4 | 1.8 ± 0.6 | 2.1 ± 0.4 | 2.5 ± 0.3 | 2.2 ± 0.4 | 1.7 ± 0.2 | 2.0 ± 0.4 | 2.4 ± 0.6 | 1.9 ± 0.6 | 1.8 ± 0.3 | 2.0 ± 0.4 | 2.2 ± 0.6 | 1.5 ± 0.2 | 1.9 ± 0.4 | 2.3 ± 0.6 |
| 20:5n-3 | 2.9 ± 0.9 | 2.5 ± 0.7 | 2.7 ± 0.7 | 3.1 ± 0.6 | 3.0 ± 0.8 | 2.2 ± 0.4 | 2.8 ± 1.0 | 3.3 ± 1.2 | 2.8 ± 1.2 | 2.2 ± 0.6 | 2.8 ± 0.9 | 2.7 ± 1.1 | 1.7 ± 0.3 | 2.8 ± 1.1 | 2.6 ± 1.2 |
| 22:5n-3 | 2.6 ± 1.3 | 2.8 ± 0.9 | 3.1 ± 1.1 | 2.8 ± 0.9 | 3.5 ± 1.2 | 2.2 ± 0.4 | 3.2 ± 1.7 | 3.4 ± 1.3 | 3.9 ± 2.2 | 2.1 ± 0.7 | 3.6 ± 1.4 | 2.3 ± 0.9 | 1.8 ± 0.5 | 3.2 ± 1.9 | 2.3 ± 1.0 |
| 22:6n-3 | 5.3 ± 1.4 | 4.6 ± 1.8 | 3.9 ± 0.9 | 5.8 ± 1.2 | 4.5 ± 1.2 | 3.3 ± 0.4 | 4.4 ± 1.6 | 6.2 ± 1.9 | 4.0 ± 1.7 | 3.2 ± 0.6 | 4.2 ± 1.6 | 4.9 ± 1.3 | 2.6 ± 0.5 | 4.1 ± 1.7 | 4.7 ± 1.5 |
| ∑n-3 | 14.3 ± 5.3 | 12.8 ± 3.8 | 12.8 ± 4.2 | 15.6 ± 4.4 | 14.5 ± 5.2 | 9.6 ± 2.2 | 13.5 ± 6.3 | 17.9 ± 7.2 | 14.2 ± 7.2 | 9.4 ± 3.0 | 13.7 ± 5.4 | 13.0 ± 5.6 | 7.3 ± 1.8 | 13.7 ± 7.1 | 12.2 ± 6.1 |
| ∑n-6 | 6.3 ± 2.4 | 5.2 ± 1.4 | 5.7 ± 2.0 | 6.2 ± 1.7 | 5.8 ± 2.1 | 4.1 ± 1.1 | 5.6 ± 2.7 | 7.2 ± 3.1 | 5.5 ± 2.5 | 4.0 ± 1.4 | 5.4 ± 2.1 | 5.4 ± 2.4 | 3.0 ± 0.9 | 5.4 ± 2.5 | 5.5 ± 3.0 |
| ***Drum*** | | **Storage at -80°C** | | | | **Ice for 3 h then storage at -20°C** | | | | **Ice for 6 h then storage at -20°C** | | | | **On ice + N_2_** | **On ice – N_2_** |
| **Fatty Acid** | **Initial** | **1 week** | **1 month** | **3 months** | **6 months** | **1 week** | **1 month** | **3 months** | **6 months** | **1 week** | **1 month** | **3 months** | **6 months** | **1 week** | **1 week** |
| 16:0 | 2.6 ± 0.2 | 2.9 ± 0.3 | 3.1 ± 0.2 | 4.2 ± 0.4 | 3.1 ± 0.1 | 2.8 ± 0.2 | 3.6 ± 0.1 | 3.6 ± 0.6 | 2.6 ± 0.2 | 2.7 ± 0.3 | 2.9 ± 0.4 | 3.3 ± 0.4 | 2.3 ± 0.4 | 2.7 ± 0.3 | 2.8 ± 0.6 |
| 16:1n-7 | 0.6 ± 0.04 | 0.7 ± 0.1 | 0.9 ± 0.1 | 1.1 ± 0.2 | 0.8 ± 0.03 | 0.7 ± 0.1 | 1.0 ± 0.1 | 1.1 ± 0.3 | 0.6 ± 0.1 | 0.7 ± 0.2 | 0.8 ± 0.2 | 0.9 ± 0.2 | 0.6 ± 0.2 | 0.6 ± 0.1 | 0.7 ± 0.2 |
| 18:0 | 0.8 ± 0.1 | 0.8 ± 0.1 | 0.9 ± 0.1 | 1.4 ± 0.3 | 0.9 ± 0.1 | 0.8 ± 0.1 | 1.1 ± 0.2 | 1.0 ± 0.3 | 0.7 ± 0.1 | 0.8 ± 0.1 | 0.8 ± 0.1 | 0.9 ± 0.2 | 0.6 ± 0.1 | 0.8 ± 0.2 | 0.7 ± 0.2 |
| 18:1n-9 | 1.2 ± 0.1 | 1.4 ± 0.3 | 1.6 ± 0.3 | 2.4 ± 0.4 | 1.4 ± 0.1 | 1.3 ± 0.2 | 1.9 ± 0.2 | 2.0 ± 0.5 | 1.1 ± 0.1 | 1.3 ± 0.3 | 1.4 ± 0.3 | 1.7 ± 0.4 | 1.1 ± 0.3 | 1.3 ± 0.2 | 1.3 ± 0.3 |
| 18:1n-7 | 0.6 ± 0.04 | 0.7 ± 0.1 | 1.8 ± 0.2 | 1.1 ± 0.1 | 0.7 ± 0.04 | 0.7 ± 0.1 | 0.8 ± 0.1 | 0.9 ± 0.2 | 0.5 ± 0.1 | 0.6 ± 0.1 | 0.7 ± 0.1 | 0.8 ± 0.2 | 0.5 ± 0.1 | 0.6 ± 0.1 | 0.7 ± 0.1 |
| 18:2n-6 | 0.3 ± 0.02 | 0.3 ± 0.1 | 0.4 ± 0.1 | 0.5 ± 0.1 | 0.3 ± 0.03 | 0.3 ± 0.03 | 0.4 ± 0.04 | 0.4 ± 0.9 | 0.2 ± 0.03 | 0.3 ± 0.04 | 0.4 ± 0.06 | 0.4 ± 0.1 | 0.3 ± 0.1 | 0.3 ± 0.04 | 0.3 ± 0.1 |
| 18:3n-3 | 0.2 ± 0.02 | 0.2 ± 0.03 | 0.3 ± 0.03 | 0.3 ± 0.3 | 0.3 ± 0.02 | 0.2 ± 0.01 | 0.3 ± 0.02 | 0.2 ± 0.1 | 0.2 ± 0.03 | 0.2 ± 0.03 | 0.3 ± 0.03 | 0.3 ± 0.03 | 0.2 ± 0.03 | 0.2 ± 0.04 | 0.2 ± 0.03 |
| 20:4n-6 | 2.4 ± 0.1 | 2.7 ± 0.4 | 2.6 ± 0.2 | 2.9 ± 0.2 | 2.6 ± 0.3 | 2.6 ± 0.2 | 2.8 ± 0.2 | 2.2 ± 0.6 | 2.0 ± 0.2 | 2.5 ± 0.1 | 2.5 ± 0.2 | 2.5 ± 0.2 | 1.8 ± 0.3 | 2.1 ± 0.3 | 2.5 ± 0.3 |
| 20:5n-3 | 2.7 ± 0.1 | 3.0 ± 0.4 | 2.8 ± 0.2 | 2.9 ± 0.2 | 2.9 ± 0.3 | 2.9 ± 0.1 | 3.0 ± 0.2 | 2.2 ± 0.8 | 2.2 ± 0.2 | 2.7 ± 0.1 | 2.8 ± 0.2 | 2.58 ± 0.2 | 1.9 ± 0.3 | 2.2 ± 0.4 | 2.8 ± 0.3 |
| 22:5n-3 | 1.0 ± 0.1 | 1.2 ± 0.1 | 1.2 ± 0.2 | 1.5 ± 0.3 | 1.1 ± 0.1 | 1.1 ± 0.1 | 1.5 ± 0.4 | 1.1 ± 0.3 | 0.8 ± 0.1 | 1.2 ± 0.1 | 1.2 ± 0.1 | 1.2 ± 0.2 | 0.8 ± 0.2 | 0.9 ± 0.2 | 1.2 ± 0.2 |
| 22:6n-3 | 0.6 ± 0.03 | 0.8 ± 0.1 | 0.8 ± 0.1 | 1.0 ± 0.2 | 0.7 ± 0.1 | 0.7 ± 0.1 | 0.8 ± 0.1 | 0.6 ± 0.2 | 0.5 ± 0.04 | 0.7 ± 0.1 | 0.7 ± 0.1 | 0.7 ± 0.1 | 0.5 ± 0.1 | 0.6 ± 0.1 | 0.7 ± 0.1 |
| ∑n-3 | 4.7 ± 0.2 | 5.3 ± 0.6 | 5.2 ± 0.5 | 6.0 ± 0.6 | 5.0 ± 0.5 | 5.0 ± 0.5 | 5.6 ± 0.6 | 4.2 ± 1.3 | 3.8 ± 0.4 | 4.9 ± 0.3 | 5.1 ± 0.4 | 4.8 ± 0.5 | 3.5 ± 0.6 | 4.1 ± 0.8 | 5.1 ± 0.6 |
| ∑n-6 | 3.0 ± 0.1 | 3.3 ± 0.5 | 3.1 ± 0.2 | 3.6 ± 0.3 | 3.2 ± 0.2 | 3.3 ± 0.2 | 3.5 ± 0.3 | 2.9 ± 1.6 | 2.5 ± 0.2 | 3.0 ± 0.2 | 3.2 ± 0.3 | 3.2 ± 0.3 | 2.3 ± 0.5 | 2.8 ± 0.5 | 5.5 ± 3.0 |
| ***Catfish*** | | **Storage at -80°C** | | | | **Ice for 3 h then storage at -20°C** | | | | **Ice for 6 h then storage at -20°C** | | | | **On ice + N_2_** | **On ice – N_2_** |
| **Fatty Acid** | **Initial** | **1 week** | **1 month** | **3 months** | **6 months** | **1 week** | **1 month** | **3 months** | **6 months** | **1 week** | **1 month** | **3 months** | **6 months** | **1 week** | **1 week** |
| 16:0 | 4.5 ± 1.1 | 4.7 ± 1.3 | 4.2 ± 1.3 | 5.4 ± 2.0 | 4.2 ± 0.2 | 6.3 ± 3.7 | 5.1 ± 1.6 | 5.3 ± 1.4 | 8.5 ± 6.1 | 4.8 ± 0.7 | 4.4 ± 1.0 | 4.8 ± 0.7 | 6.1 ± 3.2 | 3.6 ± 0.4 | 4.5 ± 1.1 |
| 16:1n-7 | 1.1 ± 0.6 | 1.1 ± 0.9 | 0.9 ± 0.8 | 1.5 ± 1.2 | 0.6 ± 0.2 | 2.1 ± 2.2 | 1.3 ± 1.0 | 1.7 ± 0.9 | 3.5 ± 3.6 | 1.2 ± 0.5 | 1.1 ± 0.6 | 1.3 ± 0.5 | 2.2 ± 1.9 | 0.5 ± 0.3 | 1.1 ± 0.7 |
| 18:0 | 1.8 ± 0.3 | 1.8 ± 0.3 | 1.6 ± 0.2 | 2.0 ± 0.6 | 1.8 ± 0.04 | 2.4 ± 0.9 | 2.0 ± 0.4 | 2.1 ± 0.4 | 2.9 ± 1.7 | 2.0 ± 0.2 | 1.8 ± 0.2 | 2.0 ± 0.2 | 2.2 ± 0.9 | 1.7 ± 0.1 | 1.9 ± 0.3 |
| 18:1n-9 | 5.6 ± 2.7 | 5.8 ± 3.6 | 4.7 ± 3.2 | 7.2 ± 4.7 | 3.9 ± 0.7 | 9.8 ± 8.9 | 6.6 ± 4.0 | 7.9 ± 3.5 | 15.4 ± 14 | 5.9 ± 1.9 | 5.7 ± 2.6 | 6.2 ± 1.8 | 10.1 ± 7.5 | 3.4 ± 1.1 | 5.6 ± 2.9 |
| 18:1n-7 | 1.5 ± 0.6 | 1.5 ± 0.7 | 1.3 ± 0.6 | 1.8 ± 1.0 | 1.1 ± 0.2 | 2.2 ± 1.8 | 1.6 ± 0.7 | 1.8 ± 0.7 | 3.2 ± 2.9 | 1.4 ± 0.4 | 1.4 ± 0.5 | 1.5 ± 0.4 | 2.3 ± 1.5 | 0.9 ± 0.2 | 1.4 ± 0.6 |
| 18:2n-6 | 0.6 ± 0.2 | 0.6 ± 0.3 | 0.5 ± 0.3 | 0.7 ± 0.4 | 0.4 ± 0.1 | 0.9 ± 0.8 | 0.7 ± 0.3 | 0.8 ± 0.3 | 1.4 ± 1.3 | 0.6 ± 0.2 | 0.6 ± 0.2 | 0.6 ± 0.2 | 1.0 ± 0.7 | 0.3 ± 0.1 | 0.6 ± 0.3 |
| 18:3n-3 | 0.3 ± 0.2 | 0.3 ± 0.2 | 0.3 ± 0.2 | 0.4 ± 0.3 | 0.2 ± 0.1 | 0.6 ± 0.6 | 0.4 ± 0.2 | 0.5 ± 0.3 | 0.9 ± 0.9 | 0.3 ± 0.1 | 0.3 ± 0.2 | 0.4 ± 0.3 | 0.6 ± 0.5 | 0.2 ± 0.1 | 0.3 ± 0.2 |
| 20:4n-6 | 2.4 ± 0.4 | 2.5 ± 0.2 | 2.4 ± 0.2 | 2.7 ± 0.3 | 2.6 ± 0.1 | 2.8 ± 0.6 | 2.7 ± 0.1 | 2.6 ± 0.2 | 3.1 ± 1.2 | 2.6 ± 0.1 | 2.4 ± 0.2 | 2.6 ± 0.1 | 2.8 ± 0.7 | 2.2 ± 0.2 | 2.3 ± 0.2 |
| 20:5n-3 | 2.1 ± 0.3 | 2.1 ± 0.2 | 2.0 ± 0.2 | 2.2 ± 0.3 | 2.1 ± 0.1 | 2.4 ± 0.6 | 2.3 ± 0.2 | 2.2 ± 0.3 | 2.7 ± 1.2 | 2.2 ± 0.1 | 2.0 ± 0.2 | 2.2 ± 0.1 | 2.4 ± 0.7 | 1.8 ± 0.2 | 2.0 ± 0.2 |
| 22:5n-3 | 0.8 ± 0.3 | 0.9 ± 0.4 | 0.7 ± 0.3 | 1.0 ± 0.5 | 0.7 ± 0.1 | 1.3 ± 1.0 | 0.9 ± 0.5 | 1.1 ± 0.4 | 1.9 ± 1.7 | 0.9 ± 0.2 | 0.8 ± 0.3 | 0.9 ± 0.2 | 1.3 ± 0.9 | 0.5 ± 0.1 | 0.8 ± 0.3 |
| 22:6n-3 | 4.1 ± 0.5 | 4.1 ± 0.2 | 4.0 ± 0.3 | 4.2 ± 0.3 | 4.3 ± 0.1 | 4.5 ± 0.8 | 4.4 ± 0.2 | 4.3 ± 0.3 | 4.8 ± 1.7 | 4.4 ± 0.1 | 4.0 ± 0.2 | 4.3 ± 0.1 | 4.5 ± 0.9 | 3.8 ± 0.3 | 4.0 ± 0.4 |
| ∑n-3 | 7.4 ± 1.2 | 7.7 ± 0.3 | 7.3 ± 0.7 | 8.0 ± 1.4 | 7.5 ± 0.2 | 9.0 ± 3.3 | 8.2 ± 1.1 | 8.2 ± 1.3 | 10.8 ± 6.0 | 8.0 ± 0.5 | 7.3 ± 0.8 | 7.9 ± 0.6 | 9.1 ± 3.2 | 6.4 ± 0.6 | 7.2 ± 1.0 |
| ∑n-6 | 3.4 ± 0.7 | 3.6 ± 0.6 | 3.4 ± 0.6 | 5.1 ± 0.9 | 3.4 ± 0.1 | 4.4 ± 1.9 | 3.8 ± 0.7 | 4.9 ± 0.6 | 5.4 ± 3.3 | 3.7 ± 0.3 | 3.4 ± 0.5 | 3.7 ± 0.3 | 4.4 ± 1.7 | 2.8 ± 0.3 | 3.3 ± 0.6 |
| ***Eelpout*** | | **Storage at -80°C** | | | | **Ice for 3 h then storage at -20°C** | | | | **Ice for 6 h then storage at -20°C** | | | | **On ice + N_2_** | **On ice – N_2_** |
| **Fatty Acid** | **Initial** | **1 week** | **1 month** | **3 months** | **6 months** | **1 week** | **1 month** | **3 months** | **6 months** | **1 week** | **1 month** | **3 months** | **6 months** | **1 week** | **1 week** |
| 16:0 | 2.6 ± 1.5 | 2.3 ± 0.8 | 1.8 ± 1.1 | 4.2 ± 1.8 | 2.3 ± 1.1 | 4.2 ± 1.7 | 2.8 ± 1.4 | 4.4 ± 1.8 | 3.2 ± 1.8 | 3.3 ± 0.4 | 2.4 ± 1.4 | 2.3 ± 0.7 | 3.3 ± 1.1 | 1.1 ± 0.4 | 3.4 ± 3.5 |
| 16:1n-7 | 4.2 ± 3.0 | 3.5 ± 0.8 | 2.6 ± 2.3 | 6.8 ± 4.0 | 3.8 ± 2.2 | 7.6 ± 3.3 | 4.5 ± 3.1 | 7.2 ± 3.2 | 5.2 ± 3.6 | 5.3 ± 0.8 | 3.7 ± 2.5 | 3.3 ± 1.3 | 5.1 ± 2.1 | 1.5 ± 0.9 | 4.3 ± 3.8 |
| 18:0 | 0.5 ± 0.3 | 0.6 ± 0.4 | 0.4 ± 0.1 | 0.9 ± 0.3 | 0.3 ± 0.1 | 0.6 ± 0.2 | 0.5 ± 0.2 | 0.8 ± 0.3 | 0.4 ± 0.2 | 0.5 ± 0.03 | 0.4 ± 0.1 | 0.4 ± 0.1 | 0.5 ± 0.1 | 0.2 ± 0.03 | 0.6 ± 0.7 |
| 18:1n-9 | 7.8 ± 5.2 | 7.0 ± 3.3 | 5.3 ± 4.2 | 12.7 ± 6.7 | 6.9 ± 3.8 | 13.7 ± 10 | 11.0 ± 6.0 | 16.7 ± 7.0 | 11.1 ± 7.2 | 11.1 ± 1.5 | 7.8 ± 5.0 | 7.2 ± 2.5 | 11.1 ± 3.8 | 3.1 ± 1.4 | 11.4 ± 11 |
| 18:1n-7 | 2.2 ± 1.6 | 2.2 ± 1.0 | 1.7 ± 1.3 | 4.0 ± 2.0 | 2.2 ± 1.2 | 3.9 ± 1.6 | 2.8 ± 1.5 | 4.3 ± 1.8 | 2.9 ± 1.8 | 2.9 ± 0.3 | 2.1 ± 1.3 | 1.9 ± 0.6 | 2.9 ± 1.0 | 1.0 ± 0.4 | 3.0 ± 2.9 |
| 18:2n-6 | 0.7 ± 0.6 | 0.7 ± 0.3 | 0.5 ± 0.4 | 1.2 ± 0.6 | 0.6 ± 0.3 | 1.0 ± 0.4 | 0.6 ± 0.4 | 1.0 ± 0.4 | 0.6 ± 0.4 | 0.6 ± 0.1 | 0.4 ± 0.3 | 0.4 ± 0.1 | 0.6 ± 0.2 | 0.3 ± 0.1 | 0.6 ± 0.6 |
| 18:3n-3 | 0.3 ± 0.3 | 0.3 ± 0.1 | 0.2 ± 0.2 | 0.6 ± 0.3 | 0.3 ± 0.2 | 0.6 ± 0.2 | 0.3 ± 0.2 | 0.5 ± 0.2 | 0.3 ± 0.2 | 0.4 ± 0.1 | 0.3 ± 0.2 | 0.2 ± 0.1 | 0.3 ± 0.1 | 0.1 ± 0.1 | 0.3 ± 0.2 |
| 20:4n-6 | 0.3 ± 0.2 | 0.2 ± 0.1 | 0.3 ± 0.1 | 0.5 ± 0.2 | 0.2 ± 0.1 | 0.5 ± 0.2 | 0.4 ± 0.1 | 0.5 ± 0.2 | 0.3 ± 0.1 | 0.4 ± 0.02 | 0.4 ± 0.1 | 0.4 ± 0.1 | 0.2 ± 0.2 | 0.2 ± 0.02 | 0.5 ± 0.5 |
| 20:5n-3 | 2.9 ± 1.8 | 2.7 ± 0.9 | 2.4 ± 1.6 | 4.9 ± 2.4 | 2.1 ± 1.1 | 6.1 ± 2.5 | 4.1 ± 2.1 | 6.4 ± 2.5 | 3.4 ± 1.9 | 4.5 ± 0.4 | 3.5 ± 1.8 | 3.3 ± 0.9 | 3.2 ± 1.0 | 1.4 ± 0.5 | 4.7 ± 4.6 |
| 22:5n-3 | 0.6 ± 0.5 | 0.7 ± 0.2 | 0.6 ± 0.3 | 1.2 ± 0.5 | 0.5 ± 0.2 | 1.5 ± 0.6 | 1.0 ± 0.5 | 1.6 ± 0.6 | 0.8 ± 0.5 | 0.5 ± 0.04 | 0.4 ± 0.2 | 0.4 ± 0.1 | 0.7 ± 0.3 | 0.4 ± 0.1 | 1.1 ± 1.1 |
| 22:6n-3 | 2.0 ± 0.9 | 2.2 ± 0.4 | 2.1 ± 0.7 | 3.4 ± 1.1 | 1.5 ± 0.5 | 4.6 ± 1.7 | 3.3 ± 1.2 | 5.0 ± 1.8 | 2.4 ± 1.1 | 3.1 ± 0.1 | 2.6 ± 1.0 | 2.6 ± 0.5 | 2.3 ± 0.6 | 1.3 ± 0.2 | 4.2 ± 4.5 |
| ∑n-3 | 6.5 ± 4.1 | 6.3 ± 1.3 | 5.8 ± 3.4 | 11.7 ± 5.1 | 5.0 ± 2.3 | 14.0 ± 5.6 | 9.6 ± 4.4 | 14.7 ± 5.7 | 7.7 ± 4.3 | 9.4 ± 0.7 | 7.4 ± 3.7 | 7.1 ± 1.8 | 7.2 ± 2.2 | 3.5 ± 1.0 | 11.0 ± 11 |
| ∑n-6 | 1.1 ± 0.1 | 1.0 ± 0.4 | 0.9 ± 0.6 | 1.9 ± 0.9 | 0.9 ± 0.5 | 1.9 ± 0.7 | 1.2 ± 0.7 | 1.8 ± 0.7 | 1.2 ± 0.7 | 1.3 ± 0.4 | 1.0 ± 0.5 | 0.9 ± 0.3 | 1.1 ± 0.4 | 0.5 ± 0.1 | 1.3 ± 1.3 |
| ***Trout*** | | **Storage at -80°C** | | | | **Ice for 3 h then storage at -20°C** | | | | **Ice for 6 h then storage at -20°C** | | | | **On ice + N_2_** | **On ice – N_2_** |
| **Fatty Acid** | **Initial** | **1 week** | **1 month** | **3 months** | **6 months** | **1 week** | **1 month** | **3 months** | **6 months** | **1 week** | **1 month** | **3 months** | **6 months** | **1 week** | **1 week** |
| 16:0 | - | 10.8 ± 4.7 | 13.4 ± 10 | 14.7 ± 8.3 | 7.0 ± 2.5 | 6.0 ± 1.2 | 9.3 ± 6.6 | 7.0 ± 1.5 | 9.9 ± 7.8 | 14.8 ± 1.9 | 16.2 ± 5.4 | 20.8 ± 14 | 22.0 ± 14 | 14.0 ± 3.9 | 20.9 ± 4.6 |
| 16:1n-7 | - | 2.3 ± 1.5 | 3.3 ± 3.3 | 2.8 ± 1.8 | 1.3 ± 0.7 | 0.9 ± 0.3 | 1.9 ± 2.1 | 1.2 ± 0.5 | 2.0 ± 2.2 | 3.1 ± 0.6 | 3.8 ± 1.7 | 5.2 ± 4.7 | 5.6 ± 4.5 | 3.1 ± 1.2 | 5.2 ± 1.5 |
| 18:0 | - | 2.2 ± 1.0 | 2.9 ± 2.2 | 2.9 ± 1.6 | 1.5 ± 0.5 | 1.3 ± 0.2 | 2.0 ± 1.4 | 1.5 ± 0.3 | 2.0 ± 1.6 | 2.9 ± 0.4 | 3.3 ± 1.1 | 4.2 ± 3.1 | 4.5 ± 3.1 | 2.8 ± 0.8 | 4.2 ± 1.0 |
| 18:1n-9 | - | 10.1 ± 6.5 | 14.7 ± 15 | 12.0 ± 7.6 | 5.7 ± 3.2 | 4.0 ± 1.3 | 8.5 ± 9.0 | 5.5 ± 2.1 | 8.8 ± 9.4 | 13.8 ± 2.6 | 16.8 ± 7.4 | 22.5 ± 21 | 24.1 ± 20 | 12.8 ± 5.2 | 21.8 ± 6.6 |
| 18:1n-7 | - | 1.3 ± 0.8 | 1.9 ± 1.8 | 1.6 ± 1.0 | 0.8 ± 0.4 | 0.6 ± 0.1 | 1.1 ± 1.1 | 0.8 ± 0.3 | 1.2 ± 1.2 | 1.8 ± 0.3 | 2.2 ± 0.9 | 2.8 ± 2.4 | 3.1 ± 2.5 | 1.7 ± 0.6 | 2.8 ± 0.8 |
| 18:2n-6 | - | 6.3 ± 4.1 | 9.2 ± 9.4 | 7.6 ± 4.8 | 3.7 ± 2.1 | 2.6 ± 0.8 | 5.4 ± 5.5 | 3.4 ± 1.3 | 5.4 ± 5.8 | 8.7 ± 1.5 | 10.1 ± 4.2 | 12.9 ± 11 | 13.8 ± 10 | 7.9 ± 3.1 | 13.3 ± 3.5 |
| 18:3n-3 | - | 1.2 ± 0.8 | 1.7 ± 1.7 | 1.5 ± 0.9 | 0.7 ± 0.4 | 0.5 ± 0.1 | 1.0 ± 1.0 | 0.7 ± 0.2 | 1.1 ± 1.1 | 1.7 ± 0.3 | 1.9 ± 0.8 | 2.4 ± 1.9 | 2.6 ± 1.9 | 1.5 ± 0.6 | 2.5 ± 0.7 |
| 20:4n-6 | - | 0.5 ± 0.1 | 0.5 ± 0.3 | 0.6 ± 0.3 | 0.3 ± 0.1 | 0.3 ± 0.01 | 0.3 ± 0.1 | 0.3 ± 0.0.4 | 0.4 ± 0.2 | 0.6 ± 0.05 | 0.6 ± 0.1 | 0.7 ± 0.4 | 0.7 ± 0.3 | 0.5 ± 0.1 | 0.7 ± 0.1 |
| 20:5n-3 | - | 2.7 ± 0.9 | 3.4 ± 2.1 | 3.8 ± 2.0 | 2.1 ± 0.5 | 1.8 ± 0.2 | 2.3 ± 1.1 | 2.0 ± 0.3 | 2.4 ± 1.3 | 3.6 ± 0.3 | 3.8 ± 1.0 | 4.5 ± 2.6 | 4.7 ± 2.4 | 3.3 ± 0.7 | 4.4 ± 0.8 |
| 22:5n-3 | - | 0.7 ± 0.4 | 1.0 ± 0.9 | 1.0 ± 0.5 | 0.5 ± 0.2 | 0.4 ± 0.04 | 0.6 ± 0.4 | 0.5 ± 0.1 | 0.6 ± 0.4 | 0.9 ± 0.1 | 1.0 ± 0.3 | 1.2 ± 0.7 | 1.3 ± 0.8 | 0.8 ± 0.2 | 1.2 ± 0.3 |
| 22:6n-3 | - | 12.4 ± 3.8 | 14.8 ± 8.0 | 17.8 ± 9.3 | 9.9 ± 2.3 | 8.9 ± 0.7 | 10.2 ± 3.7 | 9.4 ± 1.0 | 10.4 ± 4.7 | 14.9 ± 1.1 | 15.4 ± 2.9 | 17.4 ± 7.6 | 17.9 ± 6.9 | 13.9 ± 1.8 | 17.2 ± 2.5 |
| ∑n-3 | - | 19.7 ± 7.3 | 24.6 ± 16 | 27.3 ± 14 | 14.9 ± 4.1 | 12.7 ± 1.3 | 16.3 ± 8.1 | 14.1 ± 2.0 | 16.8 ± 9.6 | 24.6 ± 2.3 | 26.2 ± 6.4 | 20.7 ± 17 | 32.0 ± 16 | 22.9 ± 4.4 | 30.7 ± 5.5 |
| ∑n-6 | - | 7.1 ± 4.3 | 10.2 ± 10 | 8.7 ± 5.3 | 4.3 ± 2.3 | 3.1 ± 0.9 | 5.9 ± 5.9 | 4.0 ± 1.4 | 6.1 ± 6.2 | 9.7 ± 1.7 | 11.3 ± 3.5 | 14.4 ± 12 | 15.5 ± 11 | 8.9 ± 3.2 | 14.6 ± 3.8 |
| ***Charr*** | | **Storage at -80°C** | | | | **Ice for 3 h then storage at -20°C** | | | | **Ice for 6 h then storage at -20°C** | | | | **On ice + N_2_** | **On ice – N_2_** |
| **Fatty Acid** | **Initial** | **1 week** | **1 month** | **3 months** | **6 months** | **1 week** | **1 month** | **3 months** | **6 months** | **1 week** | **1 month** | **3 months** | **6 months** | **1 week** | **1 week** |
| 16:0 | - | 21.1 ± 3.9 | 35.4 ± 5.3 | 31.4 ± 6.1 | 17.9 ± 3.0 | 25.3 ± 7.3 | 20.0 ± 2.7 | 17.3 ± 3.4 | 37.5 ± 20 | 20.1 ± 3.3 | 25.8 ± 12 | 19.3 ± 6.7 | 11.3 ± 2.4 | 11.9 ± 2.3 | 14.0 ± 1.9 |
| 16:1n-7 | - | 7.7 ± 1.9 | 13.6 ± 2.7 | 12.2 ± 3.3 | 6.2 ± 1.5 | 9.4 ± 3.3 | 7.0 ± 1.1 | 5.7 ± 1.5 | 16.0 ± 9.8 | 7.4 ± 1.7 | 10.2 ± 5.8 | 7.0 ± 3.0 | 3.4 ± 1.1 | 3.8 ± 1.1 | 4.4 ± 0.9 |
| 18:0 | - | 4.4 ± 0.8 | 7.3 ± 1.0 | 6.6 ± 1.2 | 3.8 ± 0.7 | 5.3 ± 1.6 | 4.1 ± 0.6 | 3.7 ± 0.7 | 7.8 ± 4.1 | 4.2 ± 0.7 | 5.4 ± 2.5 | 4.0 ± 1.4 | 2.4 ± 0.5 | 2.5 ± 0.5 | 2.9 ± 0.5 |
| 18:1n-9 | - | 30.4 ± 7.4 | 52.0 ± 10 | 49.3 ± 13 | 26.0 ± 6.6 | 36.9 ± 13 | 27.7 ± 4.3 | 23.4 ± 5.3 | 63.7 ± 38 | 29.5 ± 6.6 | 40.6 ± 22 | 27.7 ± 12 | 14.2 ± 4.2 | 15.4 ± 4.3 | 18.1 ± 3.8 |
| 18:1n-7 | - | 4.6 ± 1.1 | 8.0 ± 1.4 | 7.5 ± 1.8 | 4.0 ± 0.9 | 5.5 ± 1.9 | 4.3 ± 0.6 | 3.6 ± 0.8 | 9.2 ± 5.3 | 4.4 ± 0.9 | 5.9 ± 3.2 | 4.2 ± 1.7 | 2.2 ± 0.6 | 2.4 ± 0.6 | 2.8 ± 0.6 |
| 18:2n-6 | - | 10.1 ± 2.5 | 17.3 ± 3.3 | 16.5 ± 4.3 | 8.6 ± 2.2 | 12.2 ± 4.3 | 9.1 ± 1.4 | 7.7 ± 1.8 | 21.1 ± 13 | 9.5 ± 2.1 | 12.9 ± 7.1 | 9.0 ± 4.0 | 4.6 ± 1.4 | 4.9 ± 1.5 | 5.7 ± 1.3 |
| 18:3n-3 | - | 2.5 ± 0.6 | 4.3 ± 0.8 | 4.1 ± 1.1 | 2.1 ± 0.5 | 2.8 ± 1.0 | 2.2 ± 0.3 | 1.9 ± 0.4 | 5.1 ± 3.2 | 2.3 ± 0.5 | 3.1 ± 1.7 | 2.2 ± 1.0 | 1.1 ± 0.3 | 1.2 ± 0.4 | 1.4 ± 0.3 |
| 20:4n-6 | - | 1.0 ± 0.2 | 1.4 ± 0.2 | 1.3 ± 0.2 | 0.9 ± 0.1 | 1.1 ± 0.3 | 0.9 ± 0.1 | 0.8 ± 0.1 | 1.6 ± 0.8 | 0.9 ± 0.1 | 1.2 ± 0.5 | 0.9 ± 0.3 | 0.6 ± 0.1 | 0.6 ± 0.1 | 0.7 ± 0.1 |
| 20:5n-3 | - | 8.6 ± 1.7 | 13.6 ± 2.1 | 12.9 ± 2.6 | 7.7 ± 1.5 | 10.3 ± 3.1 | 8.1 ± 1.1 | 6.9 ± 1.2 | 15.9 ± 8.2 | 8.6 ± 1.5 | 10.9 ± 5.1 | 8.0 ± 2.9 | 4.7 ± 0.9 | 5.1 ± 0.9 | 6.0 ± 1.0 |
| 22:5n-3 | - | 2.4 ± 0.5 | 3.9 ± 0.6 | 3.7 ± 0.8 | 2.2 ± 0.6 | 3.0 ± 1.0 | 2.3 ± 0.4 | 1.9 ± 0.4 | 4.8 ± 2.6 | 2.5 ± 0.5 | 3.2 ± 1.6 | 2.3 ± 1.0 | 1.3± 0.3 | 1.3 ± 0.3 | 1.7 ± 0.4 |
| 22:6n-3 | - | 20.6 ± 3.1 | 28.0 ± 3.1 | 26.7 ± 3.9 | 18.9 ± 3.3 | 23.2 ± 5.5 | 19.3 ± 2.4 | 17.4 ± 1.8 | 33.9 ± 15 | 21.7 ± 2.9 | 25.3 ± 9.3 | 19.5 ± 5.9 | 13.5 ± 1.8 | 14.4 ± 1.7 | 17.2 ± 2.5 |
| ∑n-3 | - | 39.4 ± 7.2 | 58.8 ± 8.2 | 56.1 ± 10 | 35.1 ± 7.1 | 46.0 ± 13 | 36.7 ± 5.1 | 32.3 ± 4.7 | 70.5 ± 35 | 40.3 ± 6.3 | 49.5 ± 21 | 36.8 ± 13 | 23.2 ± 4.1 | 24.7 ± 3.8 | 29.4 ± 4.9 |
| ∑n-6 | - | 11.6 ± 2.7 | 19.7 ± 3.5 | 18.7 ± 4.7 | 9.9 ± 2.5 | 14.0 ± 4.8 | 10.5 ± 1.6 | 8.9 ± 2.0 | 23.9 ± 15 | 11.0 ± 2.0 | 14.8 ± 7.9 | 10.4 ± 4.5 | 5.5 ± 1.6 | 5.8 ± 1.7 | 6.8 ± 1.5 |
