# Supplementary material for: Handling and Storage Procedures Have Variable Effects on Fatty Acid Content in Fishes with Different Lipid Quantities
Source: PLoS One. 2016 Aug 1;11(8):e0160497. doi: 10.1371/journal.pone.0160497 (PMC4968796; doi:10.1371/journal.pone.0160497)
Supplement: S2 Table — (DOCX) [file pone.0160497.s004.docx]

S2 Table. Mean and standard deviations of FAME proportions (%) of 6 fish species for all storage treatments.

| **Carp** | | **Storage at -80°C** | | | |  | **Ice for 3 h then storage at -20°C** | | | |  | **Ice for 6 h then storage at -20°C** | | | | **on ice +N_2_** | **on ice -N_2_** |
| --- | --- | --- | --- | --- | --- | --- | --- | --- | --- | --- | --- | --- | --- | --- | --- | --- | --- |
| **Fatty Acid**  16:0  16:1n7  18:0  18:1n9  18:1n7  18:2n6  18:3n3  20:4n6  20:5n3  22:5n3  22:6n3 | **Initial**  15.7±0.7  8.9±1.5  4.3±0.8  16.1±2.4  7.1±2.5  3.9±0.6  3.8±0.7  4.4±1.5  5.3±1.0  4.5±0.4  10.0±2.5 | **1 week**  15.4±0.3  8.6±0.9  4.3±0.3  16.1±1.2  7.1±0.4  4.1±0.3  3.8±0.4  3.8±0.7  5.3±0.5  5.9±1.0  9.4±1.6 | **1 month**  15.5±0.2  8.3±1.1  4.6±1.2  16.4±1.5  7.3±0.5  3.9±0.5  3.6±0.6  4.3±1.0  5.6±0.8  6.2±1.1  8.2±1.5 | **3 months**  16.0±0.3  9.2±0.8  4.0±0.2  16.5±1.2  7.8±0.3  4.1±0.4  4.0±0.5  4.4±1.3  5.3±0.9  4.6±0.5  9.9±1.8 | **6 months**  15.9±0.3  8.4±0.9  4.2±0.5  16.6±1.7  8.3±0.4  4.1±0.4  3.8±0.5  4.1±1.3  5.4±1.0  6.2±0.9*  8.2±1.6 |  | **1 week**  15.6±0.1  7.6±0.8  4.6±0.2  15.2±1.5  7.2±0.2  3.6±0.4  3.3±0.4  5.3±1.2  6.4±1.0  6.6±1.1  10.1±2.0 | **1 month**  15.4±0.4  8.2±1.9  4.1±0.4  15.5±1.8  7.2±0.3  3.9±0.5  3.6±0.7  4.7±1.9  6.0±1.4  6.4±0.7  9.7±2.4 | **3 months**  15.8±0.6  9.3±0.8  3.9±0.1  17.4±1.4  7.8±0.3  4.3±0.3  4.3±0.7  3.6±1.0  4.7±0.7  4.7±0.5  9.0±2.0 | **6 months**  16.0±0.1  8.3±0.9  4.3±0.7  16.7±1.3  8.2±0.3  4.1±0.4  3.8±0.6  3.7±1.1  5.0±0.7  6.8±1.4  7.5±1.4 |  | **1 week**  15.6±0.6  7.8±1.2  4.4±0.5  15.4±1.9  5.8±3.0  3.7±0.6  3.4±0.6  5.7±1.5  6.9±1.2  6.6±1.0  10.4±2.6 | **1 month**  15.7±0.5  7.9±1.9  4.3±1.0  15.3±3.0  7.6±1.0  3.8±0.9  3.6±1.1  4.6±1.9  5.9±1.5  7.8±3.4  9.0±2.9 | **3 months**  15.8±0.6  8.8±1.4  4.0±0.3  16.1±1.9  7.8±0.4  4.0±0.5  3.7±0.7  4.9±1.3  5.5±0.8  4.7±0.6  10.6±2.8 | **6 months**  16.3±0.9  7.0±0.9  4.8±0.8  14.4±1.3  7.9±0.4  3.4±0.5  2.9±0.5  5.9±1.1  6.5±0.9  7.0±1.8  10.3±1.6 | **1 week**  15.9±0.3  8.3±1.3  4.0±0.5  15.6±1.9  8.1±0.2  3.9±0.6  3.8±0.8  4.5±1.9  5.8±1.4  6.1±0.3  8.7±2.6 | **1 week**  16.2±0.5  8.2±2.0  4.5±0.7  15.8±3.1  5.7±3.2  3.6±0.9  3.4±1.2  5.6±1.7  6.1±1.0  5.3±1.4  11.5±2.9 |
| **Total lipid** | 9.7 ± 3.7 | 9.3 ± 3.0 | 9.0 ± 2.8 | 9.9 ± 3.0 | 10.6 ± 4.0 |  | 6.5 ± 1.6 | 9.5 ± 4.5 | 12.1 ± 5.0 | 10.1 ± 4.7 |  | 6.6 ± 2.3 | 9.3 ± 3.8 | 8.2 ± 4.0 | 5.3 ± 1.3 | 10.3 ± 5.9 | 7.6 ± 4.1 |
| **Drum** | | **Storage at -80°C** | | | |  | **Ice for 3 h then storage at -20°C** | | | |  | **Ice for 6 h then storage at -20°C** | | | | **on ice +N_2_** | **on ice -N_2_** |
| **Fatty Acid**  16:0  16:1n7  18:0  18:1n9  18:1n7  18:2n6  18:3n3  20:4n6  20:5n3  22:5n3  22:6n3 | **Initial**  18.1±0.6  4.4±0.2  5.4±0.8  8.2±0.3  4.3±0.2  2.0±0.1  1.5±0.1  17.0±0.4  19.2±0.7  7.4±0.5  4.5±0.1 | **1 week**  18.7±0.3  4.8±0.1  5.1±0.3  9.2±0.5  4.4±0.3  2.1±0.1  1.5±0.04  17.5±0.4  19.5±1.1  8.0±0.4  4.9±0.2 | **1 month**  18.9±0.7  5.2±0.2  5.5±0.4  9.7±0.6  4.8±0.5  2.3±0.1  1.6±0.1  15.7±0.7  16.9±1.2  7.4±0.4  4.8±0.4 | **3 months**  19.6±1.1  5.3±0.7  6.8±1.5  11.1±1.1  5.1±0.4  2.3±0.1  1.6±0.1  13.6±1.3  14.0±1.4  7.3±1.2  4.7±0.6 | **6 months**  19.3±0.6  4.7±0.1  5.4±0.5  8.9±0.5  4.4±0.3  2.1±0.1  1.6±0.1  16.1±0.9  17.8±1.1  6.9±0.2  4.3±0.2 |  | **1 week**  18.4±0.3  4.6±0.1  5.0±0.2  8.6±0.5  4.4±0.1  2.2±0.05  1.6±0.1  17.0±0.3  18.7±1.0  7.4±0.4  4.4±0.4 | **1 month**  19.7±0.9*  5.5±0.5*  6.0±1.2  10.3±0.2  4.7±0.5  2.2±0.1  1.5±0.1  15.5±1.0  16.6±1.1  7.9±1.3  4.6±0.4 | **3 months**  22.1±3.4  6.4±1.5  6.3±1.9  12.0±2.7  5.6±1.2  2.3±0.3  1.5±0.2  13.7±3.1  13.7±4.6  6.4±1.4  3.9±1.0 | **6 months**  20.6±1.2  4.8±0.3  5.5±0.5  9.2±0.6  4.2±0.2  2.0±0.2  1.4±0.2  16.5±0.8  18.3±1.2  6.9±0.4  4.1±0.2 |  | **1 week**  18.0±0.5  4.7±0.7  5.2±0.6  8.4±0.9  4.0±0.3  2.1±0.1  1.6±0.1  16.0±0.8  17.7±1.2  7.6±0.7  4.4±0.2 | **1 month**  18.4±0.5  4.8±0.5  5.1±0.7  8.9±0.9  4.4±0.4  2.2±0.1  1.6±0.1  15.8±0.8  17.5±1.5  7.5±0.2  4.5±0.3 | **3 months**  19.4±0.7  5.4±0.6  5.2±0.3  10.2±1.4  4.9±0.7  2.3±0.1  1.6±0.02  14.7±1.4  14.9±2.3  7.2±0.5  4.3±0.3 | **6 months**  19.7±0.5  4.9±0.6  5.2±0.3  9.1±0.5  4.2±0.3  2.2±0.1  1.6±0.05  14.8±0.5  16.3±0.8  7.0±0.4  4.2±0.3 | **1 week**  19.5±0.9  4.6±0.5  5.9±0.4  9.3±0.3  4.1±0.4  2.1±0.1  1.5±0.1  15.2±0.3  16.0±0.8  6.8±0.2  4.0±0.2 | **1 week**  17.5±1.2  4.6±0.4  4.7±0.3  8.3±0.8  4.2±0.2  2.1±0.2  1.5±0.04  15.9±0.8  17.5±1.5  7.6±0.6  4.5±0.1 |
| **Total lipid** | 3.5 ± 0.3 | 3.7 ± 0.2 | 3.6 ± 0.4 | 4.0 ± 0.3 | 3.4 ± 0.2 |  | 3.8 ± 0.2 | 4.2 ± 0.8 | 3.2 ± 0.4 | 2.5 ± 0.3 |  | 3.8 ± 0.4 | 3.6 ± 0.4 | 3.2 ± 0.4 | 2.5 ± 0.5 | 3.4 ± 0.4 | 3.7 ± 0.2 |
| **Catfish** | | **Storage at -80°C** | | | |  | **Ice for 3 h then storage at -20°C** | | | |  | **Ice for 6 h then storage at -20°C** | | | | **on ice +N_2_** | **on ice -N_2_** |
| **Fatty Acid**  16:0  16:1n7  18:0  18:1n9  18:1n7  18:2n6  18:3n3  20:4n6  20:5n3  22:5n3  22:6n3 | **Initial**  17.3±0.8  3.8±1.4  7.0±1.0  20.2±4.7  5.3±0.9  2.0±0.3  1.1±0.5  9.6±1.9  8.2±1.6  2.9±0.3  16.2±3.8 | **1 week**  16.9±0.7  3.7±1.8  6.7±1.1  19.4±5.7  5.2±0.8  2.1±0.4  1.1±0.4  9.5±2.3  8.1±1.8  3.0±0.4  15.8±4.2 | **1 month**  16.9±0.5  3.3±1.6  6.8±0.8  17.9±5.1  5.0±0.7  1.9±0.4  1.0±0.3  10.3±2.1  8.7±1.6  2.9±0.2  17.3±4.3 | **3 months**  16.4±0.1*  3.9±1.8  6.3±0.7  20.3±5.8  5.2±0.9  2.0±0.4  1.1±0.4  8.8±2.3  7.2±1.8  2.8±0.4  14.0±4.2 | **6 months**  17.7±0.4  2.7±0.6  7.5±0.4  15.6±1.8  4.7±0.4  1.8±0.1  0.9±0.2  11.0±0.9  9.1±0.6  2.8±0.1  18.5±1.7 |  | **1 week**  16.9±1.0  4.7±2.0  6.8±1.5  22.7±6.5  5.2±1.0  2.2±0.5  1.3±0.5  8.5±2.7  7.2±2.1  3.1±0.6  14.1±4.8 | **1 month**  17.0±0.4  4.1±1.4  6.7±0.6  20.7±4.8  5.0±0.6  2.1±0.3  1.2±0.3  9.6±2.1  8.1±1.7  3.0±0.4  15.7±3.8 | **3 months**  16.0±0.3*  4.7±1.4  6.3±0.9  22.7±4.0  5.2±0.7  2.2±0.3  1.3±0.4  8.2±1.7  6.8±1.1  3.1±0.4  13.5±2.9 | **6 months**  16.8±1.4  5.3±2.5  6.2±1.4  24.9±7.9  5.5±1.1  2.3±0.6  1.4±0.6  7.7±3.2  6.4±2.4  3.2±0.7  12.3±5.6 |  | **1 week**  16.8±0.6  4.1±1.1  6.9±0.7  20.4±3.6  5.0±0.5  2.1±0.3  1.2±0.3  9.5±1.6  7.9±1.3  3.0±0.3  15.7±2.9 | **1 month**  16.8±0.5  3.9±1.3  6.9±1.0  20.5±4.4  5.2±0.6  2.0±0.3  1.1±0.3  9.4±1.9  7.8±1.4  3.0±0.4  15.8±3.5 | **3 months**  16.7±0.4  4.2±1.0  6.9±0.4  21.3±2.6  5.3±0.4  2.1±0.2  1.2±0.2  9.2±1.4  7.6±0.9  3.0±0.3  15.1±2.2 | **6 months**  16.0±0.8  5.3±1.4  6.1±0.8  24.6±4.1  5.8±0.6  2.4±0.3  1.5±0.4  7.9±1.8  6.7±1.3  3.3±0.5  13.2±3.3 | **1 week**  18.0±1.0  2.6±0.9  8.3±0.9  16.4±2.9  4.5±0.4  1.7±0.3  0.8±0.2  11.2±1.2  9.2±0.8  2.4±0.3  19.2±2.1 | **1 week**  17.3±0.7  4.0±1.5  7.4±1.3  20.6±4.9  5.2±0.6  2.0±0.4  1.1±0.4  9.3±1.9  7.9±1.5  2.9±0.3  16.1±3.9 |
| **Total lipid** | 5.2 ± 0.8 | 5.0 ± 1.2 | 4.7 ± 1.0 | 5.7 ± 1.5 | 4.1 ± 0.2 |  | 6.7 ± 3.3 | 5.3 ± 1.4 | 5.8 ± 1.4 | 5.7 ± 1.9 |  | 5.3 ± 0.6 | 5.1 ± 1.0 | 5.1 ± 0.8 | 5.1 ± 0.6 | 4.4 ± 0.3 | 5.4 ± 1.2 |
| **Eelpout** | | **Storage at -80°C** | | | |  | **Ice for 3 h then storage at -20°C** | | | |  | **Ice for 6 h then storage at -20°C** | | | | **on ice +N_2_** | **on ice -N_2_** |
| **Fatty Acid**  16:0  16:1n7  18:0  18:1n9  18:1n7  18:2n6  18:3n3  20:4n6  20:5n3  22:5n3  22:6n3 | **Initial**  9.7±1.6  13.4±2.7  1.9±0.9  26.5±2.8  7.3±0.6  2.1±0.4  0.9±0.3  1.2±0.8  10.9±2.2  1.7±0.7  8.9±4.7 | **1 week**  9.1±0.8  12.8±1.8  2.4±1.7  25.8±2.0  8.2±0.5  2.5±0.3  1.2±0.1  0.8±0.2  10.4±1.0  2.6±0.3  9.1±3.0 | **1 month**  8.9±1.0  11.1±3.1  2.1±0.9  24.0±3.1  7.9±0.6  2.1±0.5  1.0±0.3  1.6±0.7  11.8±1.2  2.9±0.4  12.4±5.4 | **3 months**  9.1±1.4  13.2±2.3  2.7±2.9  25.8±1.7  8.1±0.4  2.5±0.1  1.2±0.2  1.1±0.2  10.3±0.3  2.5±0.2  8.1±1.9 | **6 months**  9.4±0.4  15.1±0.8  1.4±0.2  27.3±0.9  8.7±0.1  2.3±0.1  1.1±0.1  0.9±0.2  8.6±0.4  2.1±0.1  6.2±1.0 |  | **1 week**  8.2±1.3  14.6±3.0  1.2±0.2  27.9±2.6  7.5±0.8  2.0±0.4  1.0±0.2  1.0±0.2  11.9±2.0  2.9±0.5  9.0±1.5 | **1 month**  7.7±0.3  11.7±1.3  1.4±0.2  29.5±0.8  7.7±0.2  1.7±0.1  0.8±0.1  1.0±0.1  11.3±0.9  2.8±0.2  9.6±1.7 | **3 months**  7.7±0.2  12.4±0.5  1.4±0.6  29.1±0.5  7.4±0.1  1.7±0.1  0.8±0.03  0.9±0.03  11.1±0.2  2.8±0.04  8.8±0.4 | **6 months**  9.1±1.0  12.9±2.2  1.4±0.4  29.5±1.4  7.7±0.2  1.6±0.2  0.7±0.1  1.0±0.3  10.0±1.7  2.3±0.2  7.7±2.4 |  | **1 week**  8.6±0.03  13.9±0.5  1.3±0.1  29.1±0.5  7.5±0.2  1.6±0.05  1.0±0.03  1.2±0.1  11.7±0.4  1.3±0.04  8.3±0.7 | **1 month**  8.9±0.7  12.2±2.3  1.7±0.7  27.3±2.3  7.5±0.2  1.5±0.2  0.9±0.2  1.6±0.7  13.3±1.8  1.5±0.2  11.1±4.1 | **3 months**  9.0±0.3  12.4±1.0  1.7±0.3  27.7±0.7  7.5±0.03  1.5±0.1  0.9±0.1  1.5±0.3  12.9±0.6  1.4±0.1  10.5±1.4 | **6 months**  9.1±0.6  13.7±1.4  1.4±0.2  30.7±0.5  8.0±0.1  1.6±0.1  0.7±0.1  0.7±0.4  9.0±0.4  2.0±0.6  6.6±1.0 | **1 week**  8.4±0.8  11.1±1.8  1.5±0.4  28.4±0.5  7.5±0.1  1.6±0.2  0.7±0.1  1.2±0.2  11.6±0.7  2.8±0.1  10.3±1.9 | **1 week**  9.1±1.0  11.5±2.6  2.0±0.8  24.5±2.5  8.0±0.4  2.1±0.4  1.1±0.2  1.5±0.7  11.5±1.0  3.0±0.3  12.1±4.8 |
| **Total lipid** | 2.9 ± 2.0 | 2.6 ± 1.1 | 2.1 ± 1.5 | 5.1 ± 2.6 | 2.5 ± 1.3 |  | 5.4 ± 2.6 | 3.7 ± 2.0 | 5.7 ± 2.4 | 3.7 ± 2.3 |  | 3.8 ± 0.4 | 2.8 ± 1.7 | 2.6 ± 0.9 | 3.6 ± 1.3 | 2.1 ± 0.9 | 1.2 ± 0.5 |
| **Trout** | | **Storage at -80°C** | | | |  | **Ice for 3 h then storage at -20°C** | | | |  | **Ice for 6 h then storage at -20°C** | | | | **on ice +N_2_** | **on ice -N_2_** |
| **Fatty Acid**  16:0  16:1n7  18:0  18:1n9  18:1n7  18:2n6  18:3n3  20:4n6  20:5n3  22:5n3  22:6n3 | **Initial**  N/A  N/A  N/A  N/A  N/A  N/A  N/A  N/A  N/A  N/A  N/A | **1 week**  18.0±1.5  3.5±0.6  3.7±0.2  15.2±2.9  2.1±0.2  9.5±1.9  1.9±0.3  0.8±0.2  4.8±0.9  1.2±0.1  22.0±5.3 | **1 month**  17.9±1.7  3.6±0.7  3.7±0.2  15.9±3.1  2.1±0.3  9.9±1.9  1.9±0.3  0.8±0.2  4.6±0.8  1.2±0.02  21.0±5.1 | **3 months**  18.5±0.8  3.4±0.4  3.7±0.2  14.5±1.5  2.0±0.1  9.2±1.0  1.8±0.2  0.9±0.1  4.9±0.5  1.2±0.1  23.3±3.4 | **6 months**  17.7±0.8  3.1±0.5  3.7±0.2  13.5±2.0  1.9±0.2  8.8±1.4  1.8±0.2  0.9±0.2  5.4±0.8  1.3±0.05  26.0±4.1 |  | **1 week**  18.8±0.5  2.8±0.4  4.0±0.1  12.5±1.5  1.8±0.1  8.0±0.8  1.7±0.1  1.0±0.1  5.8±0.6  1.3±0.1  27.7±3.4 | **1 month**  18.8±1.1  3.2±0.8  3.9±0.2  14.4±3.6  2.0±0.3  9.0±2.2  1.8±0.3  0.9±0.2  5.2±1.2  1.2±0.1  23.9±6.9 | **3 months**  18.4±0.7  3.1±0.5  3.9±0.2  13.8±2.2  1.9±0.2  8.7±1.4  1.8±0.2  0.9±0.1  5.3±0.7  1.3±0.1  25.3±4.4 | **6 months**  19.0±1.0  3.2±0.9  3.8±0.2  13.9±3.7  2.0±0.3  8.6±2.3  1.8±0.3  0.9±0.3  5.4±1.3  1.2±0.2  24.4±7.7 |  | **1 week**  18.3±0.4  3.9±0.2  3.6±0.1  16.9±1.0  2.2±0.1  10.6±0.5  2.0±0.1  0.7±0.1  4.4±0.3  1.1±0.1  18.5±1.7 | **1 month**  17.8±0.5  4.0±0.5  3.6±0.1  17.8±2.0  2.3±0.2  10.8±0.9  2.0±0.1  0.7±0.1  4.3±0.5  1.1±0.1  17.8±3.5 | **3 months**  18.0±0.7  4.2±0.5  3.6±0.0  17.8±2.4  2.3±0.2  10.6±0.7  2.0±0.1  0.7±0.1  4.1±0.4  1.0±0.1  16.7±3.2 | **6 months**  18.0±0.6  4.2±0.5  3.6±0.1  18.0±2.5  2.4±0.3  10.8±0.9  2.0±0.1  0.7±0.1  4.0±0.5  1.1±0.1  16.2±3.8 | **1 week**  18.3±0.6  3.9±0.4  3.6±0.1  16.3±1.7  2.2±0.1  10.1±0.9  2.0±0.1  0.7±0.1  4.4±0.5  1.1±0.02  18.9±3.3 | **1 week**  17.7±0.3  4.4±0.2  3.5±0.02  18.3±0.8  2.3±0.1  11.2±0.2  2.1±0.04  0.6±0.04  3.8±0.2  1.0±0.03  14.8±1.2 |
| **Total lipid** | N/A | 10.3 ± 4.4 | 13.3 ± 10.2 | 10.6 ± 4.3 | 7.4 ± 2.4 |  | 6.2 ± 0.7 | 9.1 ± 6.3 | 8.3 ± 1.6 | 10.6 ± 6.9 |  | 15.6 ± 2.1 | 16.9 ± 5.1 | 17.9 ± 10.5 | 18.6 ± 9.5 | 12.2 ± 3.2 | 18.0 ± 3.5 |
| **Charr** | | **Storage at -80°C** | | | |  | **Ice for 3 h then storage at -20°C** | | | |  | **Ice for 6 h then storage at -20°C** | | | | **on ice +N_2_** | **on ice -N_2_** |
| **Fatty Acid**  16:0  16:1n7  18:0  18:1n9  18:1n7  18:2n6  18:3n3  20:4n6  20:5n3  22:5n3  22:6n3 | **Initial**  N/A  N/A  N/A  N/A  N/A  N/A  N/A  N/A  N/A  N/A  N/A | **1 week**  16.0±0.5  5.8±0.2  3.3±0.1  22.7±0.8  3.5±0.1  7.5±0.3  1.9±0.1  0.7±0.04  6.5±0.1  1.8±0.1  15.7±1.1 | **1 month**  16.3±0.2  6.2±0.2  3.4±0.1  23.7±0.6  3.6±0.04  7.9±0.2  1.9±0.04  0.7±0.03  6.3±0.1  1.8±0.02  12.9±0.6* | **3 months**  15.7±0.6  6.0±0.3  3.3±0.2  24.4±0.8  3.7±0.1  8.1±0.3  2.0±0.1  0.7±0.04  6.4±0.2  1.9±0.04  13.5±1.1 | **6 months**  15.9±0.8  5.4±0.2  3.4±0.1  22.8±0.8  3.5±0.1  7.5±0.3  1.9±0.1  0.8±0.04  6.8±0.2  1.9±0.1  16.9±1.0 |  | **1 week**  15.9±0.5  5.8±0.2  3.3±0.1  22.8±0.8  3.4±0.1  7.5±0.3  1.8±0.1  0.7±0.05  6.4±0.1  1.9±0.04  14.8±1.3 | **1 month**  16.0±0.2  5.7±0.1  3.3±0.03  22.5±0.3  3.5±0.1  7.4±0.1  1.8±0.02  0.7±0.02  6.6±0.04  1.9±0.03  15.7±0.5 | **3 months**  16.5±0.3  5.4±0.3  3.5±0.1  22.2±0.7  3.4±0.1  7.3±0.3  1.8±0.1  0.8±0.05  6.6±0.2  1.8±0.02  16.9±1.6 | **6 months**  14.9±0.5  6.1±0.4  3.1±0.1  24.4±1.2  3.6±0.1  8.0±0.6  2.0±0.1  0.7±0.1  6.4±0.4  1.9±0.05  14.1±1.9 |  | **1 week**  15.4±0.4  5.6±0.3  3.2±0.1  22.4±0.9  3.3±0.1  7.2±0.3  1.8±0.1  0.7±0.05  6.6±0.1  1.9±0.1  16.7±1.4 | **1 month**  15.4±0.8  5.8±0.7  3.2±0.2  22.9±2.2  3.4±0.2  7.3±0.7  1.8±0.2  0.7±0.1  6.5±0.4  1.9±0.1  16.1±3.7 | **3 months**  16.0±0.7  5.7±0.3  3.3±0.1  22.5±1.0  3.4±0.1  7.3±0.4  1.8±0.1  0.8±0.1  6.6±0.2  1.9±0.1  16.4±1.5 | **6 months**  16.5±0.5  4.9±0.4  3.5±0.1  20.4±1.2  3.2±0.1  6.6±0.5  1.7±0.1  0.9±0.1  6.9±0.3  1.9±0.1  20.1±2.4 | **1 week**  16.3±0.8  5.0±0.5  3.4±0.1  20.8±1.4  3.3±0.2  6.6±0.6  1.6±0.1  0.9±0.1  6.9±0.3  1.8±0.1  20.1±2.8 | **1 week**  16.2±0.7  5.1±0.2  3.4±0.1  20.8±0.7  3.2±0.1  6.6±0.4  1.6±0.1  0.8±0.05  6.9±0.1  1.9±0.1  19.9±1.1 |
| **Total lipid** | N/A | 19.3 ± 3.4 | 30.9 ± 5.4 | 29.3 ± 6.7 | 16.9 ± 2.8 |  | 24.0 ± 7.0 | 19.1 ± 2.8 | 16.2 ± 3.3 | 34.3 ± 17.6 |  | 17.0 ± 3.0 | 23.1 ± 10.6 | 17.3 ± 5.4 | 11.5 ± 2.6 | 11.7 ± 2.4 | 12.8 ± 2.0 |
